# Distribution and antimicrobial resistance patterns of urinary pathogens in preoperative midstream urine cultures from Chinese patients with urinary calculi: a meta-analysis

**DOI:** 10.1186/s12894-024-01415-w

**Published:** 2024-02-21

**Authors:** Xin Mei, Shike Zhang, Peng Xu, Zhican He, Ruizheng Tang, Baotong Yang, Iqbal Muhammad Sarfaraz, Wenqi Wu

**Affiliations:** 1 Department of Urology, The Second Affiliated Hospital of Guangzhou Medical University, Guangzhou, 510260 China; 2 Guangdong Key Laboratory of Urology, Guangzhou, 510230 China; 3 Department of Urology, The First Affiliated Hospital of Guangzhou Medical University, Guangzhou, 510230 China

**Keywords:** Urine culture, Urinary calculi, Bacterial spectrum, Antibiotic resistance, Meta-analysis

## Abstract

**Background and objective:**

This study comprehensively evaluates the distribution patterns and antimicrobial resistance profiles of urinary pathogens in Preoperative midstream urine cultures collected from patients with urinary calculi in China over the last two decades.

**Methods:**

A cross-sectional analysis of 41 studies was conducted. A systematic search across various databases, including Wanfang Data, CNKI, SinoMed, Embase, PubMed, and Web of Science, was carried out, covering the time period from 2002 to 2022. Using R 4.2.1 software, a meta-analysis was performed to assess heterogeneity using Cochran’s Q test and the I^2^ statistic.

**Results:**

In the analysis of preoperative midstream urine cultures from Chinese patients with urinary calculi, gram-negative bacteria dominated at 69%, with *Escherichia coli* (43%), *Klebsiella pneumoniae* (8%), *Proteus mirabilis* (6%), *Pseudomonas aeruginosa* (5%), *Acinetobacter baumannii* (3%), and *Enterobacter cloacae* (4%) being prominent. Gram-positive organisms included *Enterococcus faecalis* (9%), *Enterococcus faecium* (5%), and *Staphylococcus aureus* (4%). Over time, proportions of *Proteus mirabilis*, *Enterococcus faecalis*, and *Staphylococcus aureus* decreased, while *Klebsiella pneumoniae* and *Pseudomonas aeruginosa* increased. Notably, *Escherichia coli* proportion reduced from 37 to 33% within the last two decades. Antimicrobial resistance analysis indicated declining resistance in *E. coli* (e.g., co-trimoxazole from 73 to 55%, gentamicin from 64 to 40%), but rising resistance in piperacillin and cefotaxime (34–60%). *Enterococcus faecalis* exhibited increasing resistance to ampicillin (5–69%), gentamicin (59–94%), and tetracycline (77–89%) over time, while resistance to levofloxacin and ciprofloxacin notably decreased (72–16% and 49–8%, respectively).

**Conclusion:**

Over the past two decades, the proportion of gram-negative bacteria was declined, while the proportion of gram-positive bacteria increased. *Escherichia coli* remained the most common pathogen in the urine culture of patients with urinary calculi in China and the resistance of *Escherichia coli* to commonly used antibiotics increased. Clinicians should select appropriate antibiotics according to the results of urine culture and drug sensitivity test to reduce the occurrence of antibiotic resistance.

**Supplementary Information:**

The online version contains supplementary material available at 10.1186/s12894-024-01415-w.

## Introduction

Urinary lithiasis represents a prevailing condition within the domain of urology, frequently culminating in urinary obstructive complications, subsequently predisposing individuals to urinary tract infections [1]. The intricate interplay among microbial consortia within urinary milieu contributes substantively to a complex succession of events, encompassing the facilitation of urinary alkalinization, deposition of phosphate, and aggregation of crystals [2]. These intricate processes are profoundly intertwined with the underlying pathogenesis of urinary stone formation, thereby fostering the emergence of consequential clinical ramifications, such as urosepsis, systemic inflammation, and potentially, septic shock [3].

In spite of the evolving diagnostic landscape, the midstream urine culture persists as the foundational cornerstone in the clinical evaluation of urinary tract infections. Notably, while both pelvis urine culture and stone culture may yield augmented rates of positivity, their utility is confined predominantly to intraoperative settings, typified by prolonged intervals required for the determination of drug susceptibility outcomes [4, 5]. Compellingly, empirical evidence underscores the heightened vulnerability of patients harboring positive preoperative midstream urine cultures to postoperative infectious sequelae, marked by heightened occurrences of fever, systemic inflammatory response syndrome (SIRS), and sepsis, in stark contrast to their counterparts with negative urine cultures [6].

In view of these considerations, the imperative of comprehensively elucidating the intricate composition of the urinary microbiota, coupled with unraveling the intricate tapestry of antibiotic resistance patterns displayed by uropathogens in individuals afflicted by urinary calculi, becomes unequivocal. The ramifications of this understanding extend broadly, encompassing judicious antibiotic deployment, adept perioperative infection management, and the attenuation of the likelihood of calculus recurrence [7].

Despite the notable paucity of comprehensive nationwide cross-sectional investigations delineating the panorama of pathogen distribution and the landscape of drug resistance in midstream urine cultures of urinary calculus patients, a wealth of pertinent literature emanates from both domestic and international spheres. Against this backdrop, the current study undertakes a systematic analytical voyage, probing the microbial panorama and discerning the susceptibility profiles to antibiotics of pathogens resident within preoperative midstream urine cultures among patients afflicted by urinary calculi over the course of the preceding two decades in China. Through this endeavor, our aspiration is to furnish the clinical milieu with a pivotal reference, conducive to the judicious and standardized application of antibiotics, thereby yielding a salutary impact upon optimal clinical practices.

## Methods

### Literature searching protocol

The research was conducted according to the Preferred Reporting Items for Systematic Reviews and Meta-Analyses (PRISMA) statement and the meta-analysis protocol was registered on the PROSPERO database on June 3, 2023 (CRD42023428298). A systematic and meticulous approach was employed for conducting a comprehensive literature search, with the objective of retrieving pertinent studies elucidating the distribution patterns and drug resistance profiles of pathogens present in preoperative midstream urine cultures from individuals afflicted with urinary calculi. This methodological strategy entailed a thorough exploration of esteemed academic databases, including Wanfang Data, Chinese Journal Full-text Database (CNKI), SinoMed, Embase, PubMed, and Web of Science. The search encompassed studies published within the time frame spanning January 2002 to December 2022, effectively covering a duration of two decades. Our formulated search queries were thoughtfully tailored to incorporate pivotal terminologies such as “urinary calculus,” “urolithiasis,” “urinary stone,” “bacterial distribution,” “microbial spectrum,” “antibiotic sensitivity,” “drug resistance,” “bacterial culture,” and “midstream urine culture.” It is noteworthy that unpublished studies were intentionally omitted to safeguard the scholarly integrity inherent in our findings. This methodologically robust approach was directed at identifying studies that offer insights into the intricate interplay between pathogens and urinary calculi, thereby enriching our comprehension of antibiotic resistance trends and microbial dynamics within this specific contextual domain.

### Inclusion and exclusion criteria

To ensure the quality and reliability of our study, we established strict inclusion and exclusion criteria. In this meta-analysis, we included research that closely examined changes in urine culture and drug resistance patterns among individuals with urinary stones from 2002 to 2022. These studies had to focus exclusively on confirmed cases of urinary calculi, use a specific research design, collect preoperative midstream urine samples, follow consistent bacteriological culturing methods, and provide comprehensive and clear information on bacterial diversity and drug resistance outcomes, and the type of all studies was cross-sectional study. On the other hand, we excluded studies with incomplete or repetitive data, as well as abstracts or reviews that didn’t align with our research focus. Additionally, research involving participants from outside China, using different culture techniques, or relying on animal experiments were also excluded.

### Data extraction

The data extraction process was independently conducted by two researchers by screening the titles and abstracts according to the inclusion and exclusion criteria, and irrelevant literatures were excluded. The main extracted information included: name of the first author, publication year, study period during which the data were collected, study location, total number of bacterial culture specimens, number of bacteria of each genus, and drug susceptibility results.

### Quality assessment

Ensuring the integrity of our research, we meticulously evaluated the quality of the studies integrated into our analysis. Given their inherent cross-sectional design, we turned to the esteemed quality assessment criteria endorsed by The Agency for Healthcare Research and Quality (AHRQ) for observational research. Through the formulation of a tailored set of four distinct criteria, each study underwent rigorous scrutiny: (1) Precision in defining research subjects and establishing transparent inclusion/exclusion parameters; (2) Comprehensive elucidation of drug susceptibility testing methods, leaving no room for ambiguity; (3) Transparent documentation of evidence related to drug resistance patterns; (4) Achievement of sequencing outcomes surpassing the robust threshold of 70%. Assigning points to each criterion, with an aggregate score of three or more indicating a high level of methodological quality, we independently assessed the studies using a standardized data collection tool. Any discrepancies or potential biases were meticulously addressed through collaborative discussions, often involving a third researcher. This collective effort enhanced the overall robustness of our quality assessment, thus reinforcing the credibility of our findings.

### Statistical analysis

In the context of this Meta-analysis, the statistical analysis was conducted using the R 4.2.1 software. The assessment of heterogeneity among the included studies was executed through the implementation of Cochran’s Q test and subsequent computation of the I^2^ statistic. In cases where the I^2^ statistic exceeded the established threshold of 50%, indicating substantial heterogeneity, a random-effects model was employed to calculate combined rates. Given the extensive time span covered by the scrutinized literature and the potential variability in terms of publication and research durations, a strategic approach involving subgroup analyses was undertaken. These analytical subdivisions were guided by the identification of median time periods derived from the literature itself. This nuanced methodology facilitated the provision of a more comprehensive perspective that transcended mere chronological considerations.

## Results

### Search results and study characteristics

**Fig. 1:**
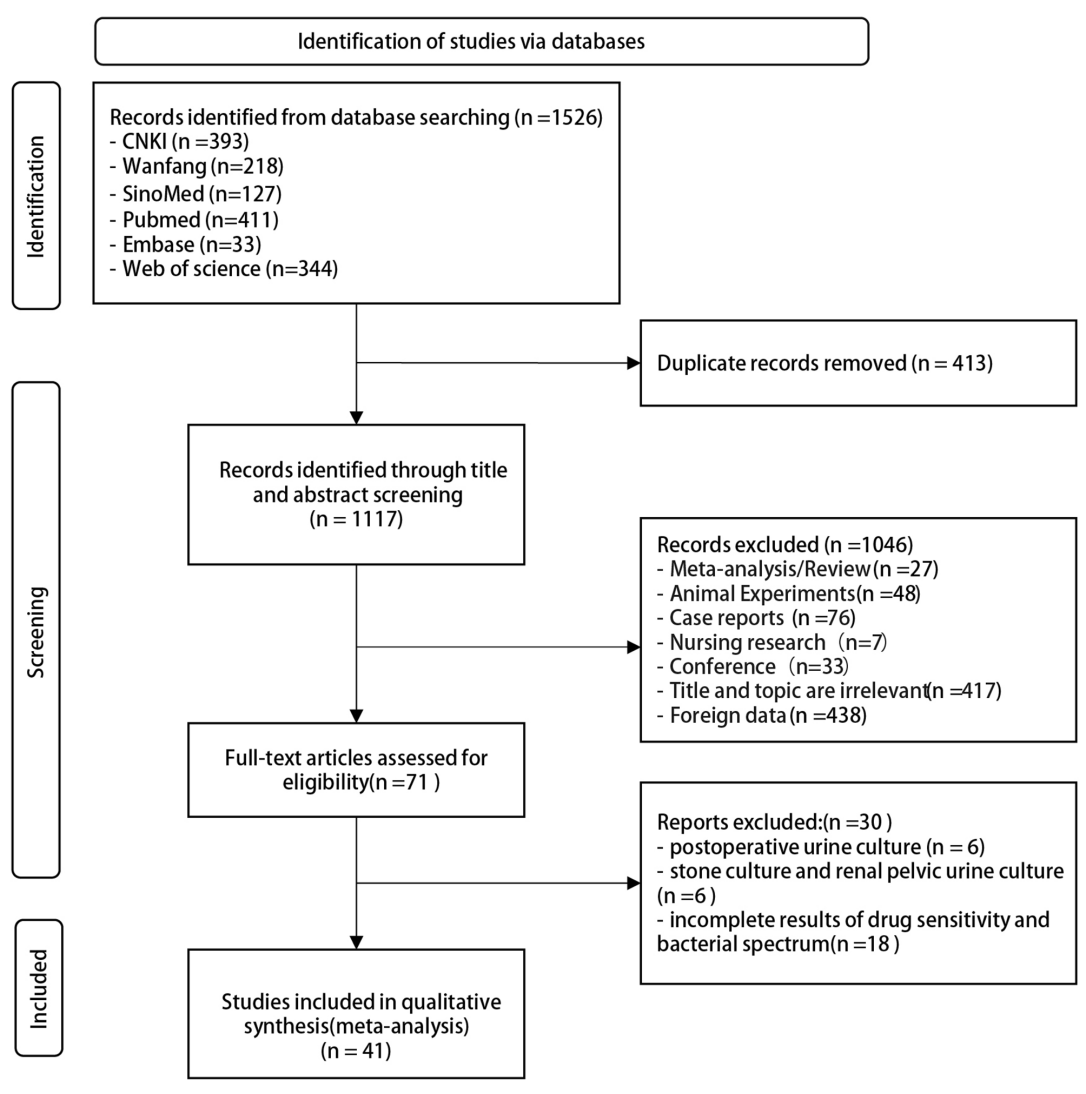
PRISMA 2020 flow diagram for systematic reviews

Initially, 3,321 relevant articles were identified. After excluding 733 duplicates, the titles and abstracts of the remaining 2,588 articles were comprehensively reviewed. Among them, 71 articles were thematically aligned with the research focus. Upon detailed examination of the full texts, 6 articles pertained to postoperative urine culture, 6 articles addressed stone culture and renal pelvic urine culture, while 18 articles had incomplete outcomes for drug sensitivity and bacterial spectrum. Subsequently, 41 cross-sectional studies [6, 8–47] meeting strict inclusion criteria were included in the meta-analysis—35 in Chinese and 6 in English. Figure 1 illustrates the literature retrieval process.

The studies included in this analysis uniformly adopted a cross-sectional study design and demonstrated quality scores exceeding 3 points. These studies were published between the years 2002 and 2022. Among them, two studies were conducted during the period of 2002 to 2007, with one each in the regions of Guangdong and Jiangxi. Shifting to the timeframe of 2008 to 2012, a total of six studies emerged, encompassing two from Zhejiang and one each from Guizhou, Anhui, Tianjin, and Yunnan. Subsequently, between 2013 and 2017, a cohort of 21 studies were published. Among these, three originated from each of Guangdong, Xizang, and Beijing, while two each emerged from Zhejiang. Additionally, there was one study from Shandong, Henan, Shaanxi, Shenzhen, Guizhou, Chongqing, Jiangxi, Hunan, Hebei, and Jiangsu. Finally, within the period of 2018 to 2022, 12 studies were published. This subset comprised three from Hunan, two each from Jiangxi and Hebei, and one each from Guizhou, Shenzhen, Henan, Fujian, and Shaanxi. In total, the studies collectively examined 17,555 pathogenic strains. Further insights into the characteristics of each individual study are available in Table 1.

**Table 1:** Basic information of included studies

NO.	Author	Publication year	Research time period	Region	Bacterial culture			Score
					Case(n)	G- (n)	G+(n)	
1	Huang JK et al. [8]	2006	2002–2005	Guangdong	453	273	147	4
2	Huang JY et al. [9]	2008	2006–2007	Jiangxi	406	348	49	4
3	He CH et al. [10]	2017	2009–2016	Guangdong	3280	2268	563	4
4	Ju GW et al. [11]	2014	2010–2013	Zhejiang	132	82	50	3
5	Xu SX et al. [12]	2015	2011–2013	Guizhou	568	391	128	4
6	Hong X et al. [13]	2014	2011–2013	Zhejiang	238	159	72	4
7	Huang JB et al. [14]	2014	2011–2013	Yunnan	416	288	94	4
8	Zhu M et al. [15]	2017	2012–2015	Henan	320	272	44	4
9	Liu XL et al. [16]	2014	2012–2013	Tianjin	58	44	13	3
10	Li J et al. [17]	2015	2013–2014	Shandong	226	151	50	3
11	Silang JC et al. [18]	2018	2015–2017	Tibet	103	73	30	3
12	Zhu CL et al. [19]	2018	2015–2018	Tibet	160	124	24	4
13	Xiao N et al. [20]	2018	2015–2017	Beijing	660	413	247	4
14	Li H et al. [21]	2017	2015–2016	Shenzhen	284	195	64	3
15	Yi SF et al. [22]	2019	2015–2017	Tibet	198	164	34	3
16	Li Y et al. [23]	2019	2015–2018	Beijing	100	66	31	4
17	Li BG et al. [24]	2018	2015–2017	Guizhou	90	63	21	3
18	Li J et al. [25]	2018	2016–2018	Jiangxi	60	37	15	3
19	Zhao WH et al. [26]	2022	2017–2020	Shaanxi	133	98	31	3
20	Yang J et al. [27]	2019	2017–2018	Guizhou	273	170	67	4
21	Zhang ZB et al. [28]	2021	2018–2019	Jiangxi	108	64	33	4
22	Liu LZ et al. [29]	2020	2018–2019	Hunan	130	79	45	3
23	Zhou H et al. [30]	2021	2019–2020	Jiangxi	79	54	14	3
24	Ao J et al. [31]	2021	2019–2020	Henan	176	106	54	4
25	Chen D et al. [32]	2018	2010–2015	Guangdong	3892	2818	694	4
26	Cui et al. [33]	2021	2015–2019	Hebei	407	344	46	4
27	Gu J et al. [34]	2022	2014–2021	Hunan	542	386	103	4
28	Wang S et al. [35]	2020	2014–2018	Beijing	457	401	140	4
29	Yang Z et al. [6]	2022	2014–2020	Fujian	141	88	46	4
30	Bai Y et al. [36]	2020	2014–2018	Hunan	353	209	69	4
31	Zhang HF et al. [37]	2019	2016–2018	Jiangsu	90	70	20	3
32	Cao RL et al. [38]	2020	2017–2018	Shenzhen	243	162	62	3
33	Liu JJ et al. [39]	2016	2014–2015	Guangdong	127	76	49	4
34	Quan KL et al. [40]	2020	2019–2019	Hunan	368	231	75	4
35	Shang XT et al. [41]	2017	2014–2016	Shaanxi	113	95	18	4
36	Wang Y et al. [42]	2015	2010–2014	Anhui	716	369	99	4
37	Wang D et al. [43]	2018	2015–2017	Chongqing	201	124	27	3
38	Xu YY et al. [44]	2019	2014–2016	Zhejiang	60	42	18	3
39	Ye JB et al. [45]	2021	2015–2019	Zhejiang	480	290	131	4
40	Yang C et al. [46]	2022	2018–2021	Hebei	600	416	136	4
41	Cui HJ et al. [47]	2022	2017–2020	Hebei	114	78	32	4

G- = Gram- negative bacteria, G+ = Gram-positive bacteria, Due to the long-time span in the literature research, the inconsistent publication time and the research time period, the median time period based on the literature research was used, rather than the publication date

### Characteristics of pathogen distribution

**Fig. 2:**
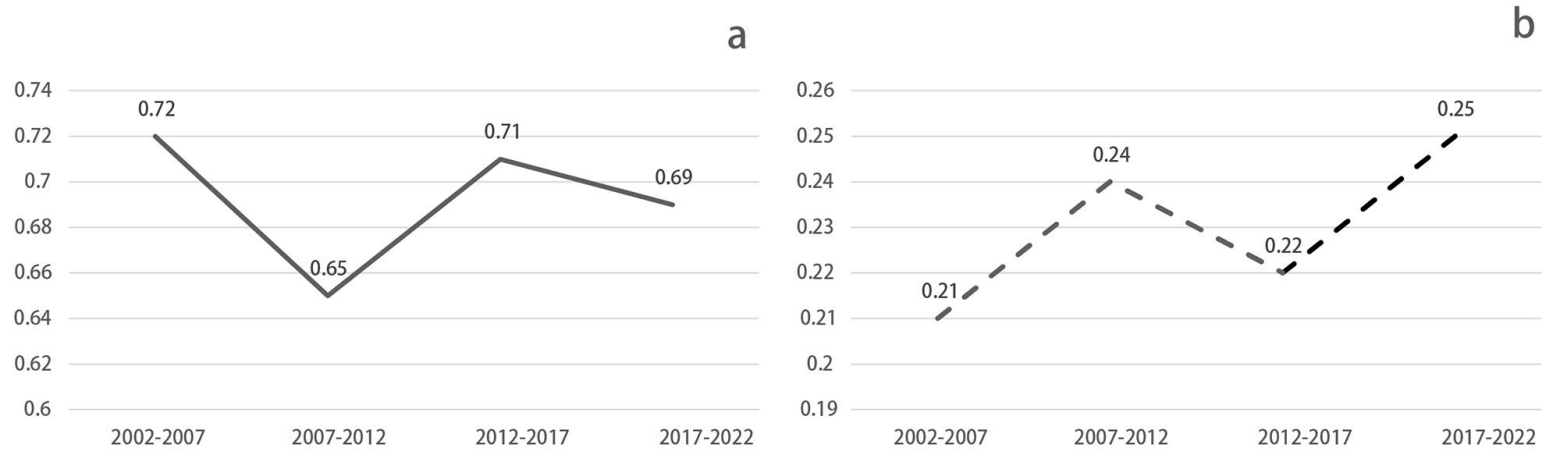
Variation pattern of the percentage of bacteria with time **a**: gram-negative bacteria **b**: gram-positive bacteria

The meta-analysis findings indicated that Chinese individuals with urinary calculi undergoing preoperative midstream urine cultures predominantly showed gram-negative bacteria, making up 69% (95%CI: 0.66–0.72) of cases, while gram-positive bacteria constituted 23% (95%CI: 0.21–0.26). Subgroup analysis displayed a declining trend in gram-negative bacteria over the past two decades (see Fig. 2).

Further genus-level analysis indicated that *Escherichia coli* was the predominant gram-negative pathogen (43%), followed by *Klebsiella pneumoniae* (8%), *Proteus mirabilis* (6%), *Pseudomonas aeruginosa* (5%), *Acinetobacter baumannii* (3%), and *Enterobacter cloacae* (4%). Among gram-positive bacteria, the key pathogens were *Enterococcus faecalis* (9%), *Enterococcus faecium* (5%), and *Staphylococcus aureus* (4%) (Table 2). From 2002 to 2022, there was a decline in *Proteus mirabilis* (7–5%), *Enterococcus faecalis* (13–9%), and *Staphylococcus aureus* (7–3%). Conversely, *Klebsiella pneumoniae* (6–10%) and *Pseudomonas aeruginosa* (3–8%) increased. Notably, *Escherichia coli* detection initially rose and then fell, dropping from 37% (95%CI: 0.09–0.71) to 33% (95%CI: 0.28–0.39) over two decades. Also, *Enterococcus faecium* rates varied between 4% (95%CI: 0.02–0.08) and 6% (95%CI: 0.04–0.07), while *Acinetobacter baumannii* (2-3%) and *Enterobacter cloacae* (3-4%) remained relatively stable (Table 2 - Annual variation of genus proportions).

**Table 2:** Annual variation of the proportion of each genus

Pathogens	2002–2007	2007–2012	2012–2017	2017–2022	I^2^	*P* value	N%(95%CI)
*Escherichia coli*	37%	43%	49%	33%	94%	<0.01	43% (0.39–0.47)
*Klebsiella pneumoniae*	8%	6%	8%	10%	68%	<0.01	8% (0.07–0.09)
*Pseudomonas aeruginosa*	4%	3%	5%	8%	83%	<0.01	5% (0.04–0.06)
*Proteus mirabilis*	7%	6%	5%	5%	81%	<0.01	6% (0.05–0.07)
*Acinetobacter baumannii*	2%	3%	2%	3%	78%	<0.01	3% (0.02–0.04)
*Enterobacter cloacae*	4%	3%	3%	4%	58%	<0.01	4% (0.03–0.05)
*Enterococcus faecalis*	13%	9%	10%	9%	80%	<0.01	9% (0.08–0.11)
*Enterococcus faecium*	/	4%	4%	6%	81%	<0.01	5% (0.04–0.05)
*Staphylococcus aureus*	7%	5%	3%	3%	88%	<0.01	4% (0.03–0.05)

N% = the combined rate of each genus in the last two decades, CI = confidence interval

### Pathogen drug resistance characteristics

In terms of common gram-negative bacteria, both *Escherichia coli* and *Klebsiella pneumoniae* displayed considerable resistance rates exceeding 80% against ampicillin. Moreover, resistance rates surpassing 50% were observed for co-trimoxazole, cefazolin, and piperacillin. However, these pathogens remained susceptible to amikacin, cefoperazone/sulbactam, and piperacillin/tazobactam, with resistance rates consistently remaining below 20%. Notably, minimal resistance was observed for imipenem, as *Escherichia coli* and *Klebsiella pneumoniae* exhibited rates of 2% (95%CI: 0.00-0.05) and 1% (95%CI: 0.00-0.03), respectively. Overall, *Escherichia coli* demonstrated a higher antibiotic resistance rate compared to *Klebsiella pneumoniae* (Table 3).

**Table 3:** The antibiotics resistance rates of *Escherichia coli* and *Klebsiella pneumoniae*

Gram- negative bacteria	*Escherichia coli*				*Klebsiella pneumoniae*			
	resistance rate(95%CI)	I^2^	*P* value	NO. of study	resistance rate(95%CI)	I^2^	*P* value	NO. of study
Ampicillin	81%(0.72–0.89)	97%	<0.01	22	94%(0.88–0.99)	87%	<0.01	16
Ampicillin/Sulbactam	53%(0.45–0.61)	94%	<0.01	14	43%(0.27–0.59)	98%	<0.01	11
Piperacillin	66%(0.52–0.78)	95%	<0.01	12	57%(0.43–0.70)	85%	<0.01	10
Piperacillin/Tazobactam	05%(0.03–0.08)	90%	<0.01	17	10%(0.05–0.16)	69%	<0.01	14
Cefazolin	69%(0.63–0.76)	94%	<0.01	19	60%(0.51–0.68)	68%	<0.01	15
Cefuroxime sodium	55%(0.48–0.63)	93%	<0.01	10	45%(0.33–0.60)	89%	<0.01	10
Cefoxitin	20%(0.16–0.25)	81%	<0.01	9	27%(0.23–0.30)	54%	0.03	8
Ceftazidime	35%(0.30–0.41)	90%	<0.01	22	36%(0.27–0.45)	74%	<0.01	18
Ceftriaxone	51%(0.44–0.58)	90%	<0.01	17	49%(0.37–0.60)	87%	<0.01	15
Cefotaxime	54%(0.46–0.63)	94%	<0.01	14	45%(0.34–0.56)	84%	<0.01	15
Cefoperazone/Sulbactam	11%(0.06–0.15)	98%	<0.01	12	15%(0.07–0.26)	69%	<0.01	11
Cefepime	31%(0.22–0.42)	97%	<0.01	16	26%(0.15–0.39)	81%	<0.01	12
Amikacin	6%(0.03–0.10)	89%	<0.01	16	14%(0.09–0.22)	75%	<0.01	14
Gentamicin	47%(0.41–0.53)	89%	<0.01	20	34%(0.26–0.43)	74%	<0.01	17
Ciprofloxacin	55%(0.47–0.63)	95%	<0.01	19	42%(0.31–0.53)	75%	<0.01	16
Levofloxacin	57%(0.50–0.64)	95%	<0.01	22	37%(0.29–0.44)	59%	<0.01	18
Aztreonam	43%(0.39–0.47)	69%	<0.01	10	37%(0.34–0.41)	0%	0.61	8
Imipenem	2%(0.00-0.05)	94%	<0.01	25	1%(0.00-0.03)	35%	<0.01	18
Co-trimoxazole	62%(0.52–0.71)	96%	<0.01	14	58%(0.44–0.72)	91%	<0.01	15

CI = confidence interval

**Fig. 3:**
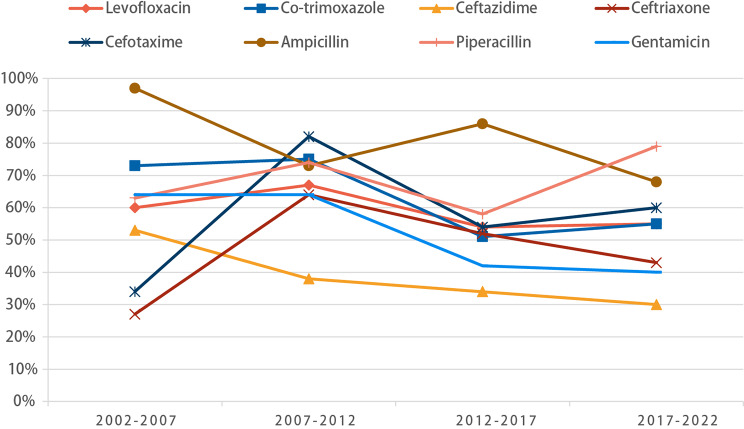
Variation pattern of the resistance rates of Escherichia coli to each antibiotic with years

Subsequent subgroup analyses revealed a gradual reduction in *Escherichia coli*’s resistance rates to various antibiotics in the study, encompassing ampicillin, gentamicin, levofloxacin, co-trimoxazole, and ceftazidime. Particularly noteworthy was the evident decline in the co-trimoxazole resistance rate, which decreased from an initial 73% (95%CI: 0.45–0.94) to a more recent 55% (95%CI: 0.46–0.63), and levofloxacin resistance decreased from 60% (95%CI: 0.49–0.70) to 55% (95%CI: 0.43–0.64) over the last two decades, however, both of them consistently remained more than 50%. Additionally, resistance to gentamicin dropped substantially from 64% (95%CI: 0.55–0.74) to 40% (95%CI: 0.35–0.45). A similar trend was observed for ceftriaxone, where resistance decreased from 64% (95%CI: 0.45–0.81) to 43% (95%CI: 0.32–0.54). In contrast, both piperacillin and cefotaxime exhibited a sustained upward trajectory. Piperacillin’s resistance rate increased from 63% (95%CI: 0.51–0.74) to 79% (95%CI: 0.70–0.86), and cefotaxime notably rose from 34% (95%CI: 0.04–0.65) to 60% (95%CI: 0.41–0.79) (Fig. 3).

### Variation in antibiotic resistance of gram-positive bacteria

In the context of common gram-positive, *Enterococcus faecalis* displayed a consistent upward trajectory in resistance rates to ampicillin, gentamicin, and tetracycline. Moreover, both *Enterococcus faecalis* and *Enterococcus faecium* exhibited notable resistance rates surpassing 70% against gentamicin and erythromycin; nevertheless, their resistance levels towards linezolid and vancomycin were notably minimal, approaching 0%. *Enterococcus faecalis* showed relative sensitivity to penicillin (29%, 95%CI: 0.11–0.52), levofloxacin (38%, 95%CI: 0.23–0.55), and ciprofloxacin (22%, 95%CI: 0.33–0.70).

**Fig. 4:**
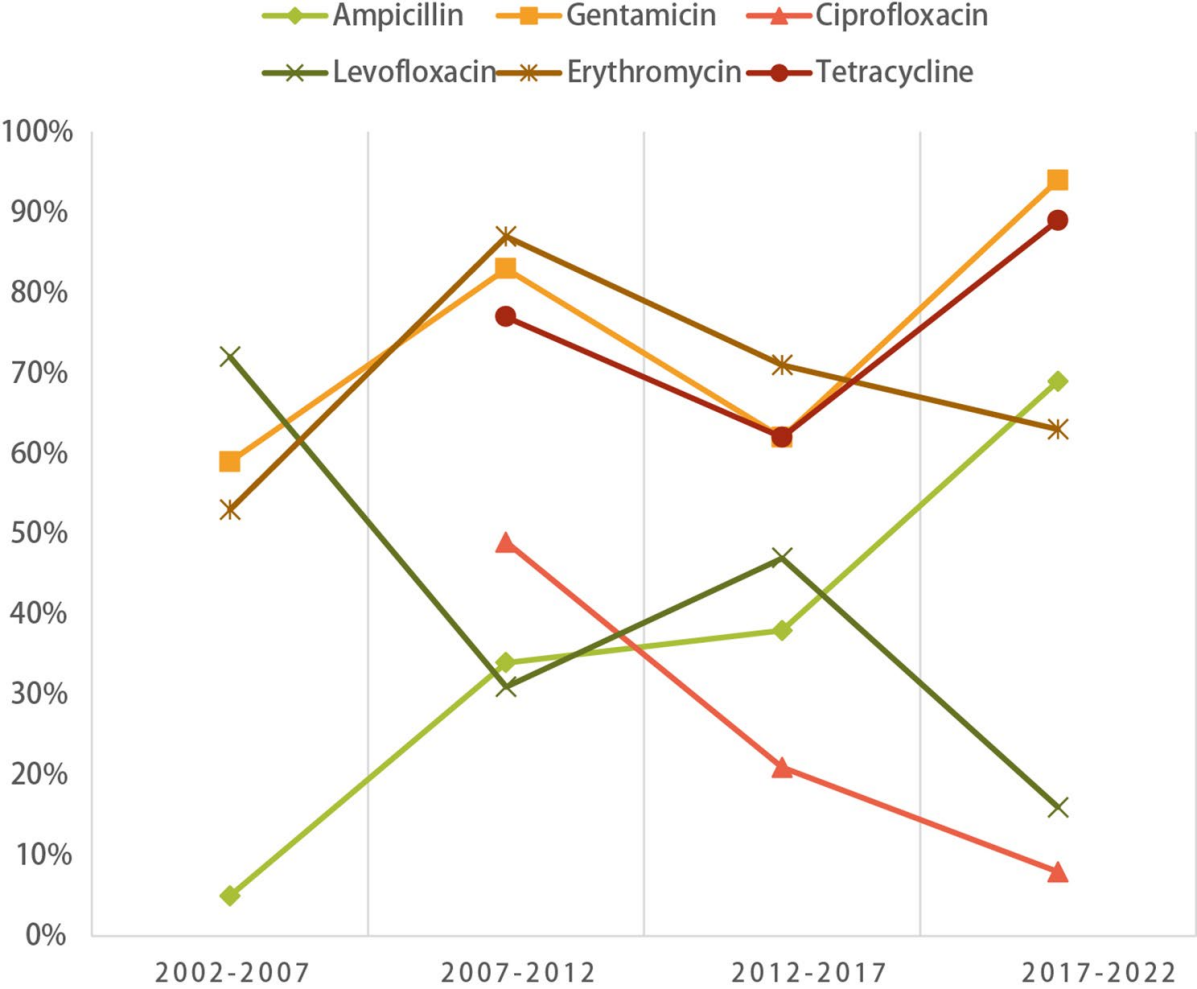
Variation pattern of the resistance rates of *Enterococcus faecalis* to each antibiotic with years

In contrast, *Enterococcus faecium* primarily displayed lower resistance to tetracycline (48%, 95%CI: 0.33–0.70), while other antibiotic resistance rates exceeded 60% (refer to Table 4). Notably, the resistance rate to ampicillin had substantially increased from 5% (95%CI: 0.01–0.14) to 69% (95%CI: 0.14-1.00), alongside a significant surge in the gentamicin resistance rate from 59% (95%CI: 0.45–0.71) to 94% (95%CI: 0.14-1.00), reaching its zenith over a two-decade span. Conversely, the resistance rate to levofloxacin had declined from 72% (95%CI: 0.59–0.83) to 16% (95%CI: 0.08–0.25), and the ciprofloxacin resistance rate had dropped from 49% (95%CI: 0.32–0.33) to 8% (95%CI: 0.00-0.33), showcasing significant recent reductions (Fig. 4).

**Table 4:** The antibiotics resistance rates of *Enterococcus faecalis* and *Enterococcus faecium*

Gram-positive bacteria	*Enterococcus faecalis*				*Enterococcus faecium*			
	Resistance rate(95%CI)	I^2^	*P* value	NO. of study	Resistance rate(95%CI)	I^2^	*P* value	NO. of study
Ampicillin	0.42(0.21–0.65)	0.97	<0.01	20	0.62(0.32–0.88)	94%	<0.01	13
Penicillin	0.29(0.11–0.52)	0.96	<0.01	19	0.80(0.53–0.97)	95%	<0.01	11
Gentamicin	0.72(0.57–0.85)	0.95	<0.01	16	0.71(0.53–0.87)	90%	<0.01	10
Ciprofloxacin	0.22(0.13–0.33)	0.83	<0.01	17	0.81(0.60–0.95)	89%	<0.01	11
Levofloxacin	0.38(0.23–0.55)	0.93	<0.01	17	0.93(0.80–0.99)	78%	<0.01	11
Erythromycin	0.72(0.62–0.81)	0.74	<0.01	18	0.84(0.63–0.97)	86%	<0.01	13
Tetracycline	0.74(0.63–0.84)	0.7	<0.01	12	0.48(0.33–0.70)	88%	<0.01	8
Linezolid	0.00(0.00-0.02)	0.39	0.08	12	0.00(0.00-0.01)	0%	0.7	7
Vancomycin	0.00(0.00-0.02)	0.57	<0.01	20	0.00(0.00-0.01)	0%	0.6	13
Teicoplanin	0.00(0.00-0.04)	0.83	<0.01	11	N	N	N	N

N = not applicable, CI = confidence interval

## Discussion

Examining complex interactions between individuals with urinary calculi, commonly called kidney stones, and their heightened susceptibility to urinary tract infections (UTIs), highlights a vital concern in the medical field. This exploration gains significance in assessing infection risks post lithotripsy, a key medical procedure posing a notable challenge. This prompts diverse diagnostic methods, including urine sample analysis before surgery, culture examination from various urinary segments, and stone scrutiny [48].

Earlier studies emphasized detecting infections via renal pelvis and stone cultures over midstream urine samples. However, concerns arise due to delayed antibiotic administration linked to time-consuming techniques. Thus, midstream urine culture analysis remains the primary UTI diagnostic route [49]. Through this meta-analysis, we explored evolving pathogenic bacteria and antibiotic resistance in Chinese urinary calculus patients (2002–2022). Findings underscore Gram-negative bacteria prevalence in midstream urine cultures over two decades. Notably, *Escherichia coli* and *Klebsiella pneumoniae* dominated Gram-negative bacteria, while *Enterococcus faecalis* and *Enterococcus faecium* were primary Gram-positive species. *Klebsiella pneumoniae* and *Pseudomonas aeruginosa* proportions grew, with a minor *Enterococcus faecalis* decreased. And the proportion of gram-negative bacteria declined, while gram-positive bacteria increased. These changes were basically consistent with the trends of bacterial spectrum monitored on China Antimicrobial Surveillance Network (CHINET) from 2018 to 2022 [50], which may be attributed to the continuous improvement of the antimicrobial management system in China [51].

The distribution and antibiotic resistance profiles of uropathogens among urinary calculus patients exhibited discrepancies due to uneven antibiotic practices and regional disparities. Our previous investigation in Guangdong province unveiled prominent trends. In this study, the most common uropathogens included *Escherichia coli* (48.7%), *Klebsiella pneumoniae* (10.4%), *Enterococcus faecalis* (8.7%), and *Proteus mirabilis* (5.2%). Importantly, among female patients, proportions of *Escherichia coli* (60.8%) and *Proteus mirabilis* (7.5%) were higher than in males, signifying gender-based microbial distinctions [32]. Another inquiry underscored that *Escherichia coli* and *Enterococcus faecalis* prevailed as the major bacteria, with a higher prevalence of *Escherichia coli* in females (53.2%) compared to males (26.6%). Furthermore, *Klebsiella pneumoniae* was more prevalent among elderly patients (9.6%) than younger ones (4.7%), while *Enterococcus faecium* exhibited a parallel trend. Importantly, *Escherichia coli* and *Klebsiella pneumoniae* exhibited susceptibility to piperacillin/tazobactam, imipenem, and amikacin (over 70%), while demonstrating resistance to penicillin, tetracycline, and vancomycin. Notably, the resistance rates of pathogens to antibiotics in male patients were significantly higher than that in female patients [52]. In addition, pathogens in older patients also showed more resistant to antibiotics than younger patients [34].

In this study we identified *Escherichia coli* as the predominant pathogen in midstream urine cultures of urinary calculus patients (43%), followed by *Enterococcus faecalis* (9%), *Klebsiella pneumoniae* (8%), *Proteus mirabilis* (6%), *Pseudomonas aeruginosa* (5%), *Enterococcus faecium* (5%), *Enterobacter cloacae* (4%), *Staphylococcus aureus* (4%), and *Acinetobacter baumannii* (3%). Similarly, a 2020 European multicenter study revealed *Escherichia coli* (41.3%) as the leading pathogen, followed by gram-positive bacteria (25.1%), KES (*Klebsiella spp*., *Enterobacter spp*., *Serratia spp*.) (14.2%), *Proteus spp*. (11.7%), and *Pseudomonas aeruginosa* (4.1%). Resistance rates to various antibiotics were notably high for quinolones, cephalosporins, and TMP/SMX, while Escherichia coli exhibited resistance rates below 10% to carbapenems, piperacillin/tazobactam, and amikacin, congruent with our findings [53]. Conversely, a U.S. single-center study reported contrasting findings, with Staphylococcus (22.2%) as the dominant pathogen in preoperative urine cultures, trailed by *Proteus* species (15.3%), *Escherichia coli* (13.1%), and *Enterococcus* (8.8%) [3].

The recent emergence of multidrug-resistant bacteria has raised significant public health concerns [35]. A comprehensive study conducted in 2019 estimated that bacterial antimicrobial resistance (AMR) contributed to around 495 million deaths, with approximately 127 million directly attributed to bacterial AMR. Notably, drug-resistant *Escherichia coli* was identified as the primary lethal pathogen, followed by *Staphylococcus aureus*, *Klebsiella pneumoniae*, *Streptococcus pneumoniae*, *Acinetobacter baumannii*, and *Pseudomonas aeruginosa* [54].

Varying economic and healthcare circumstances among nations lead to distinct patterns of bacterial distribution and drug resistance [55]. Interestingly, some lower- and middle-income countries exhibit higher rates of antibiotic resistance compared to wealthier nations, despite the former’s lower per-capita antibiotic usage. This discrepancy is largely due to the inappropriate and excessive use of antibiotics [56]. Thus, enhancing the surveillance of pathogen distribution and drug resistance is crucial to prevent the dissemination of resistant strains and curb the rise of multidrug-resistant bacteria.

This pioneering study aims to elucidate the taxonomy and pharmacological resistance of pathogenic bacteria in Chinese urinary calculi patients on a national scale. The research uncovers intricate distribution patterns and reactivity to pharmaceutical agents through preoperative midstream urinary cultures, offering valuable clinical guidance on antibiotic usage. Despite its merits, certain limitations affect the scope and applicability of the findings. The assembled literature’s quality varies due to predominant Chinese sources. Efforts to stratify studies still yield dissimilarities, partly due to literature volume. Limited inclusion of studies from 2002 to 2007 (only two) hampers understanding shifts in bacterial distribution during this period. Varying proportions of bacterial strains and resistance tendencies across institutions introduce complexity due to differing patient cohort sizes. Geographical and temporal factors also influence results, potentially causing geographic and temporal biases. Despite comprehensive scope, a focus on specific locales may create geographic bias, while absent nationwide studies introduce temporal bias. In summary, this study advances our knowledge of harmful bacteria and resistance in Chinese urinary stones patients, but limitations must be addressed for future research reliability.

## Conclusion

Over the past 20 years in China, the proportion of gram-negative bacteria was on the decline, while the proportion of gram-positive bacteria increased. *Escherichia coli* remained the most common pathogen in the urine culture of patients with urinary calculi in China, and the resistance of *Escherichia coli* to commonly used antibiotics increased, such as resistance to piperacillin and cefotaxime exhibited a gradual upward trend. Clinicians should select appropriate antibiotics according to the results of urine culture and drug sensitivity test to reduce the occurrence of antibiotic resistance. Furthermore, it is significant to strengthen the monitoring of the distribution and drug resistance of pathogens in patients with urinary calculi to prevent resistant strains increasing.

### Electronic supplementary material

Below is the link to the electronic supplementary material.

Supplementary Material 1

Supplementary Material 2

Supplementary Material 3

Supplementary Material 4

Supplementary Material 5

Supplementary Material 6

Supplementary Material 7

Supplementary Material 8

Supplementary Material 9

Supplementary Material 10

Supplementary Material 11

Supplementary Material 12

Supplementary Material 13

Supplementary Material 14

Supplementary Material 15

Supplementary Material 16

Supplementary Material 17

Supplementary Material 18

Supplementary Material 19

Supplementary Material 20

Supplementary Material 21

Supplementary Material 22

Supplementary Material 23

Supplementary Material 24

Supplementary Material 25

Supplementary Material 26

## Abbreviations

E. coli: Escherichia coli
SIRS: Systemic inflammatory response syndrome
CNKI: Chinese Journal Full-text Database
AHRQ: The Agency for Healthcare Research and Quality
CI: Confidence interval
UTIs: Urinary tract infections
CHINET: China Antimicrobial Surveillance Network
AMR: Antimicrobial resistance
