# Supplementary material for: Distribution and antimicrobial resistance patterns of urinary pathogens in preoperative midstream urine cultures from Chinese patients with urinary calculi: a meta-analysis
Source: BMC Urol. 2024 Feb 21;24:46. doi: 10.1186/s12894-024-01415-w (PMC10882938; doi:10.1186/s12894-024-01415-w)
Supplement: Supplementary file 1 — Supplementary Material 1 [file 12894_2024_1415_MOESM1_ESM.docx]

Additional file 1: Forest plot of the proportion of gram-negative bacteria with time.

Additional file 2: Forest plot of the proportion of Escherichia coli with time.

Additional file 3: Forest plot of the proportion of Klebsiella pneumoniae with time.

Additional file 4: Forest plot of the proportion of Pseudomonas aeruginosa with time.

Additional file 5: Forest plot of the proportion of Proteus mirabilis with time.

Additional file 6: Forest plot of the proportion of Acinetobacter baumannii with time.

Additional file 7: Forest plot of the proportion of Enterobacter cloacae with time.

Additional file 8: Forest plot of the proportion of gram-positive bacteria with time.

Additional file 9: Forest plot of the proportion of Enterococcus faecalis with time.

Additional file 10: Forest plot of the proportion of Enterococcus faecium with time.

Additional file 11: Forest plot of the proportion of Staphylococcus aureus with time.

Additional file 12: Forest plot of the resistance rate of Escherichia coli to Ampicillin with time.

Additional file 13: Forest plot of the resistance rate of Escherichia coli to Cefotaxime with time.

Additional file 14: Forest plot of the resistance rate of Escherichia coli to Ceftazidime with time.

Additional file 15: Forest plot of the resistance rate of Escherichia coli to Ceftriaxone with time.

Additional file 16: Forest plot of the resistance rate of Escherichia coli to Co-trimoxazole with time.

Additional file 17: Forest plot of the resistance rate of Escherichia coli to Gentamicin with time

Additional file 18: Forest plot of the resistance rate of Escherichia coli to Levofloxacin with time.

Additional file 19: Forest plot of the resistance rate of Escherichia coli to Piperacillin with time

Additional file 20: Forest plot of the resistance rate of Enterococcus faecalis to Ampicillin with time.

Additional file 21: Forest plot of the resistance rate of Enterococcus faecalis to Ciprofloxacin with time.

Additional file 22: Forest plot of the resistance rate of Enterococcus faecalis to Erythromycin with time.

Additional file 23: Forest plot of the resistance rate of Enterococcus faecalis to Gentamicin with time.

Additional file 24: Forest plot of the resistance rate of Enterococcus faecalis to Levofloxacin with time.

Additional file 25: Forest plot of the resistance rate of Enterococcus faecalis to Tetracycline with time.
