# Supplementary figures and images for: Distribution and antimicrobial resistance patterns of urinary pathogens in preoperative midstream urine cultures from Chinese patients with urinary calculi: a meta-analysis
Source: BMC Urol. 2024 Feb 21;24:46. doi: 10.1186/s12894-024-01415-w (PMC10882938; doi:10.1186/s12894-024-01415-w)

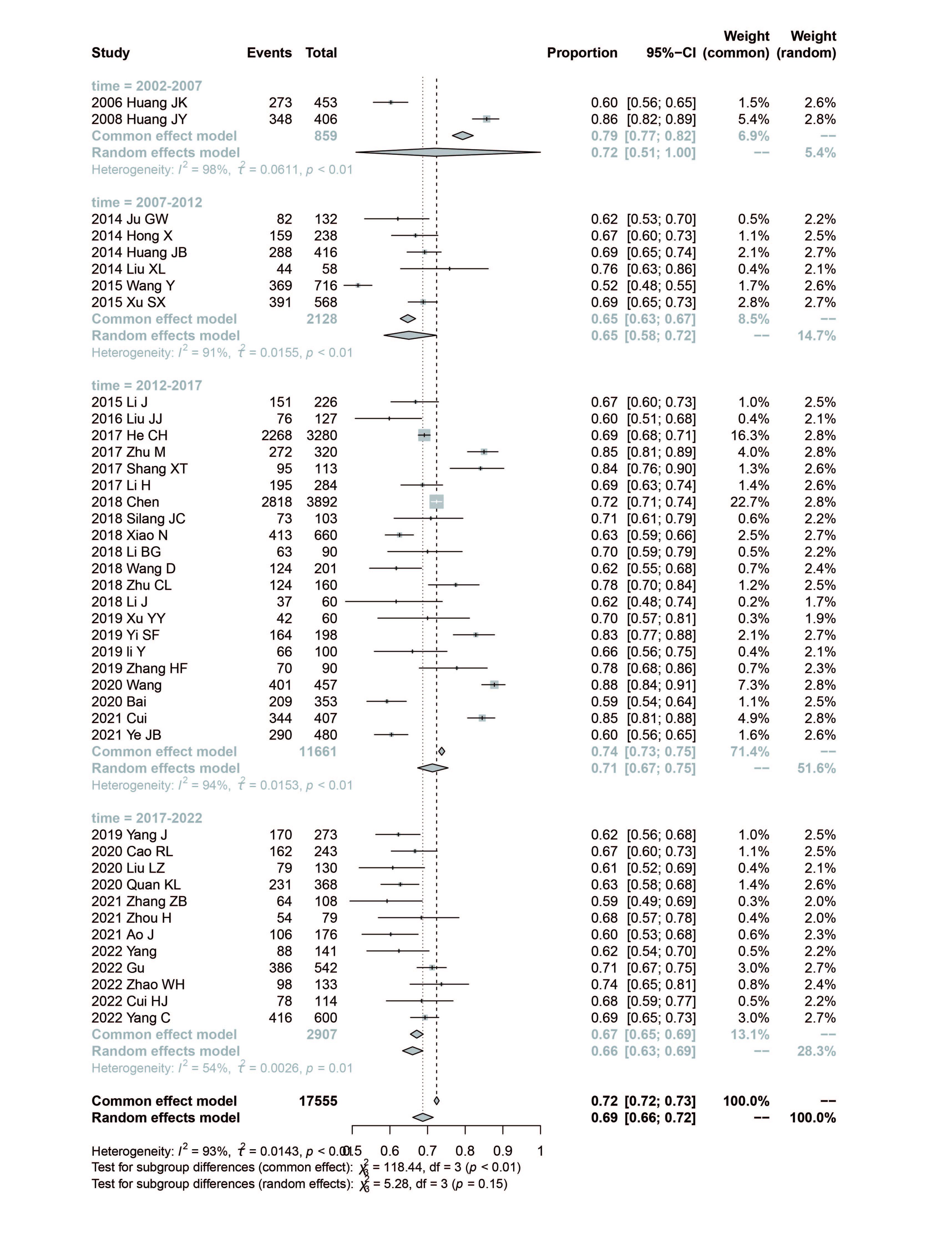

Supplement: Supplementary file 2 — Supplementary Material 2 [file 12894_2024_1415_MOESM2_ESM.jpg]

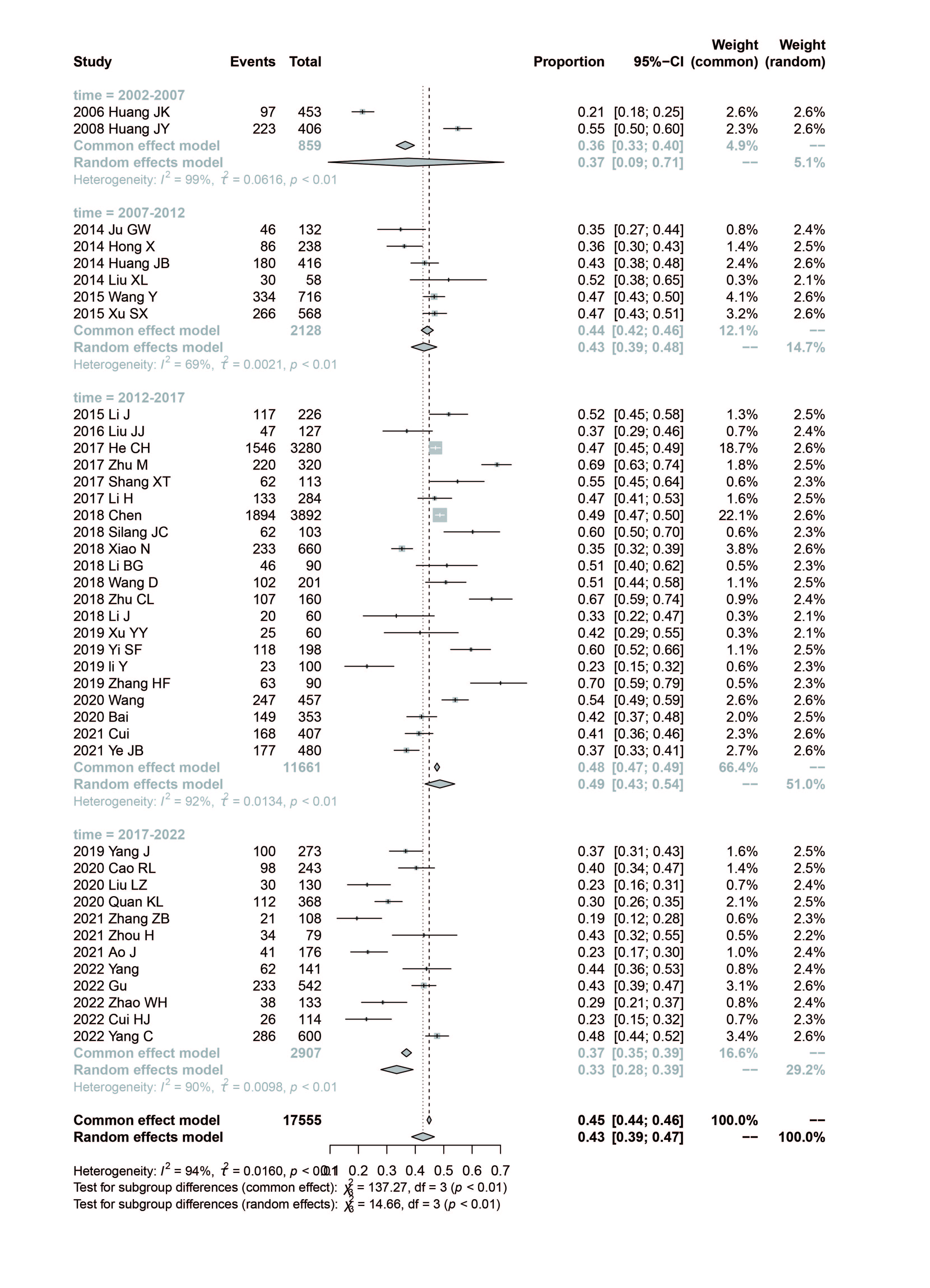

Supplement: Supplementary file 3 — Supplementary Material 3 [file 12894_2024_1415_MOESM3_ESM.jpg]

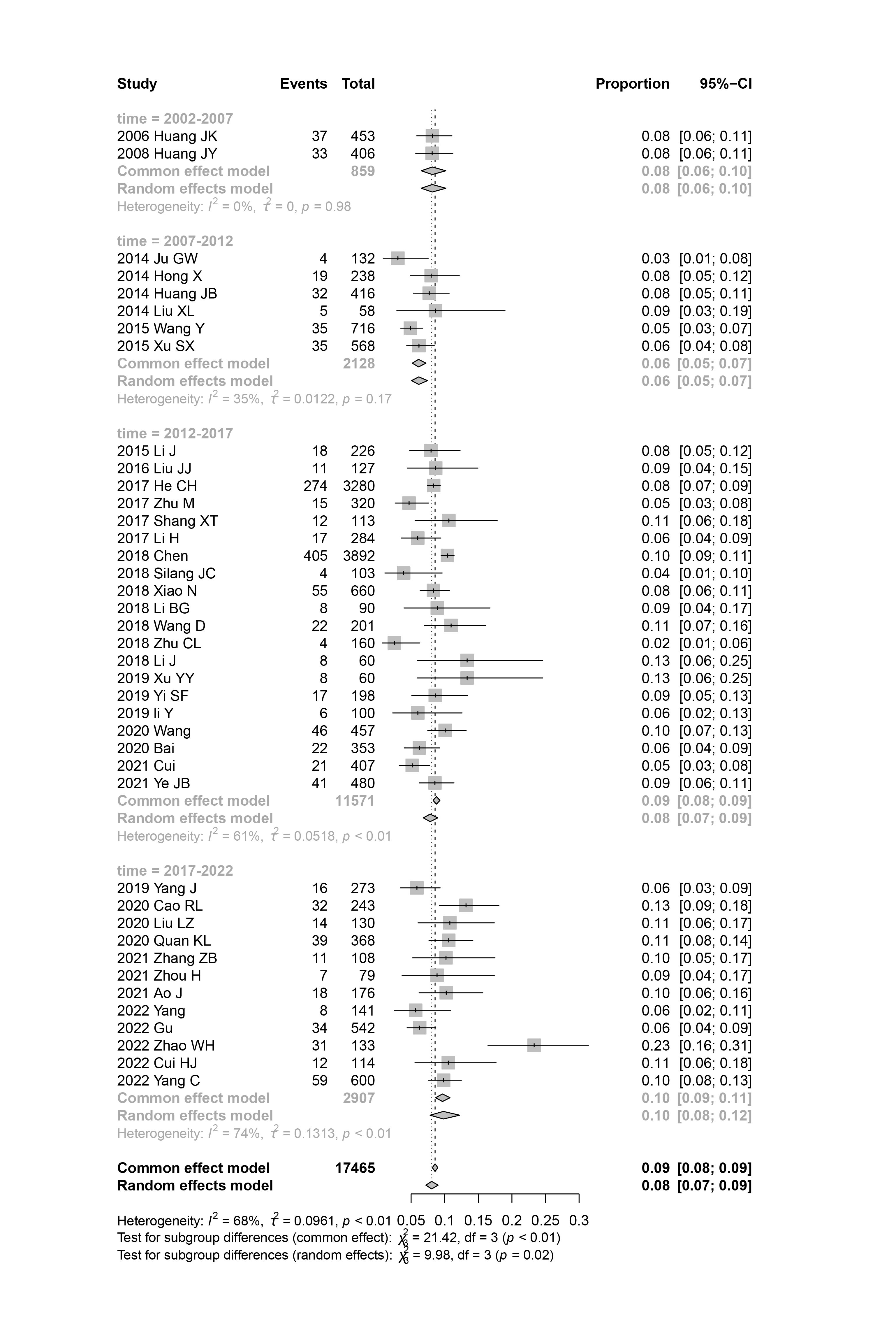

Supplement: Supplementary file 4 — Supplementary Material 4 [file 12894_2024_1415_MOESM4_ESM.jpg]

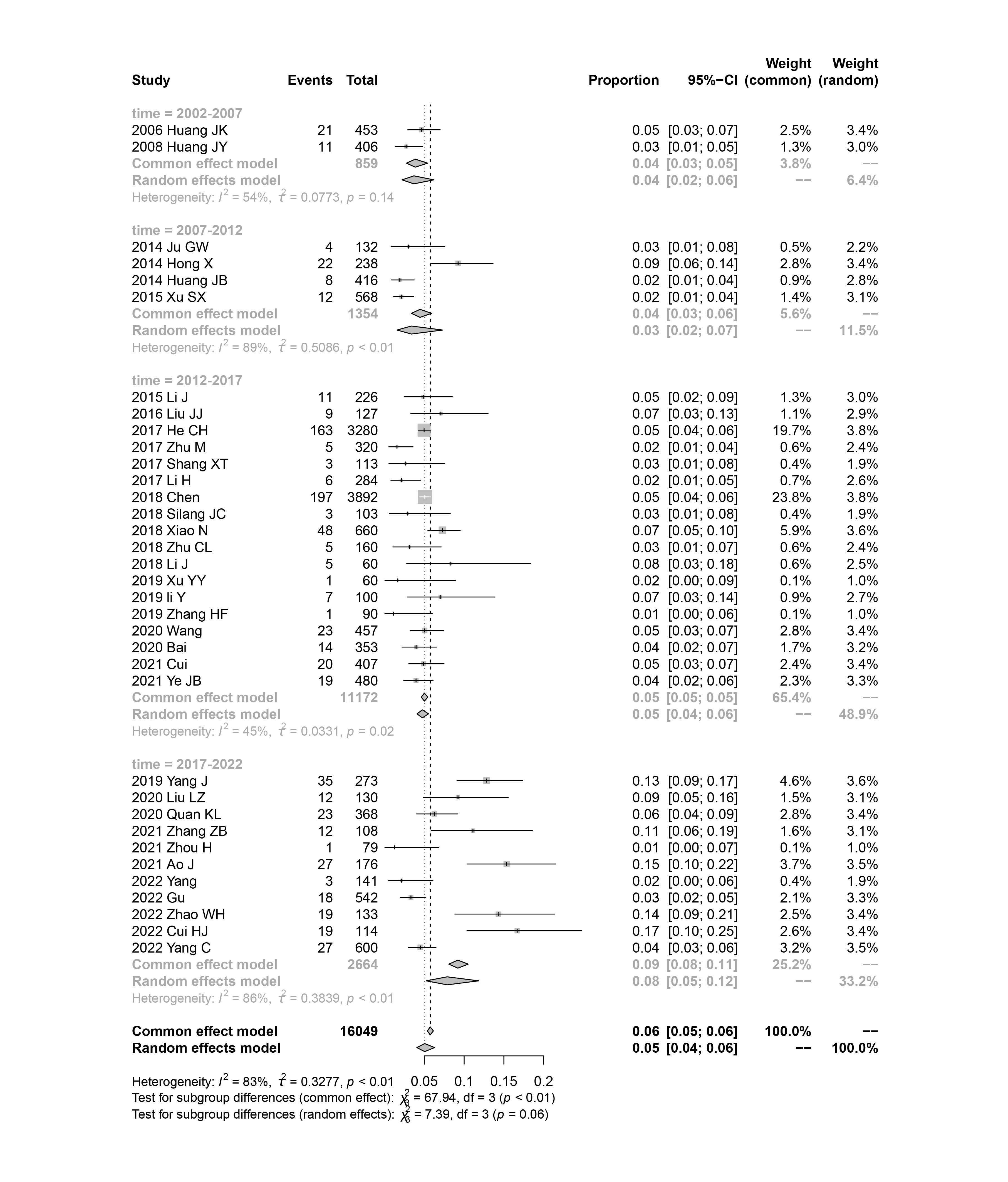

Supplement: Supplementary file 5 — Supplementary Material 5 [file 12894_2024_1415_MOESM5_ESM.jpg]

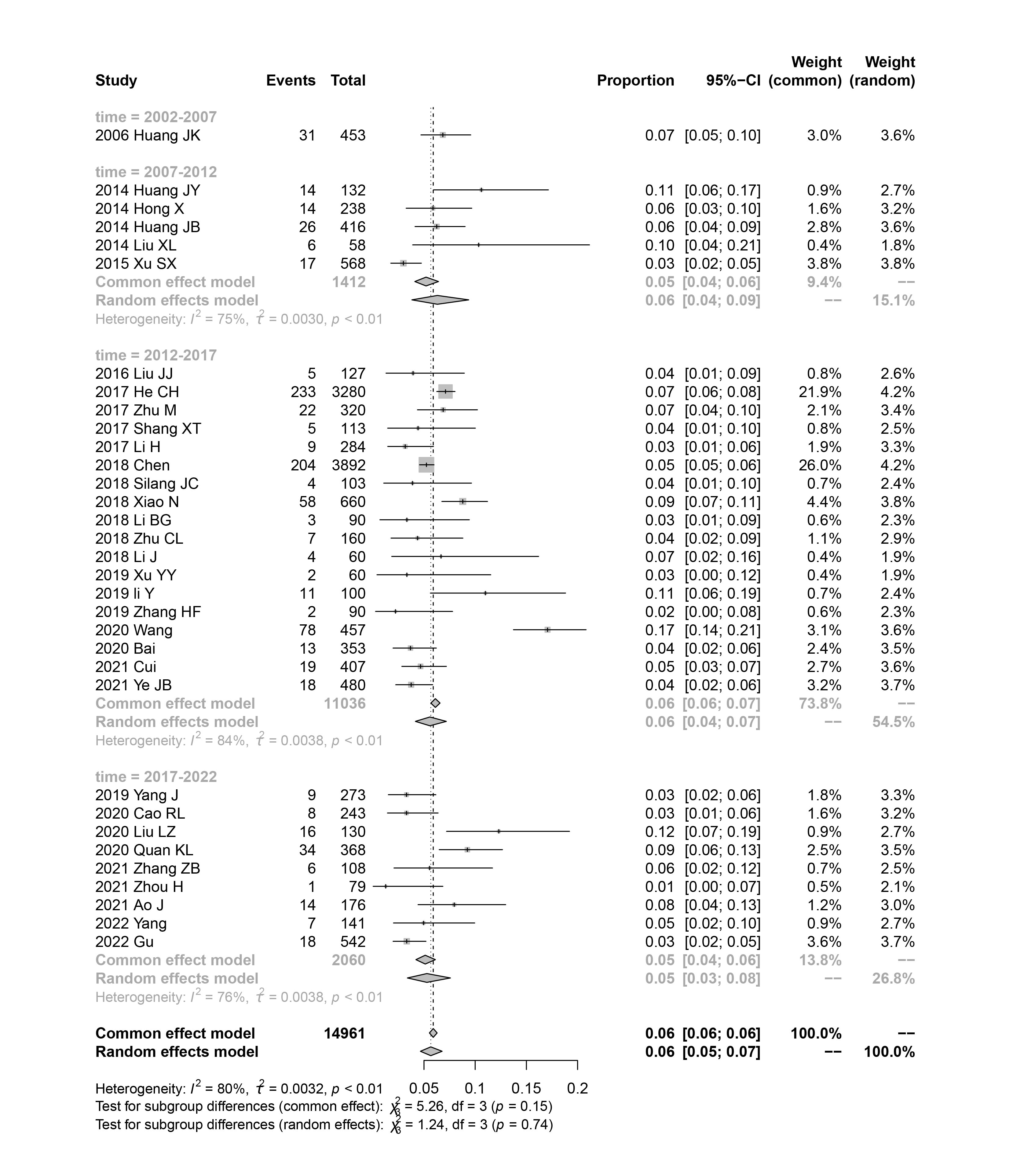

Supplement: Supplementary file 6 — Supplementary Material 6 [file 12894_2024_1415_MOESM6_ESM.jpg]

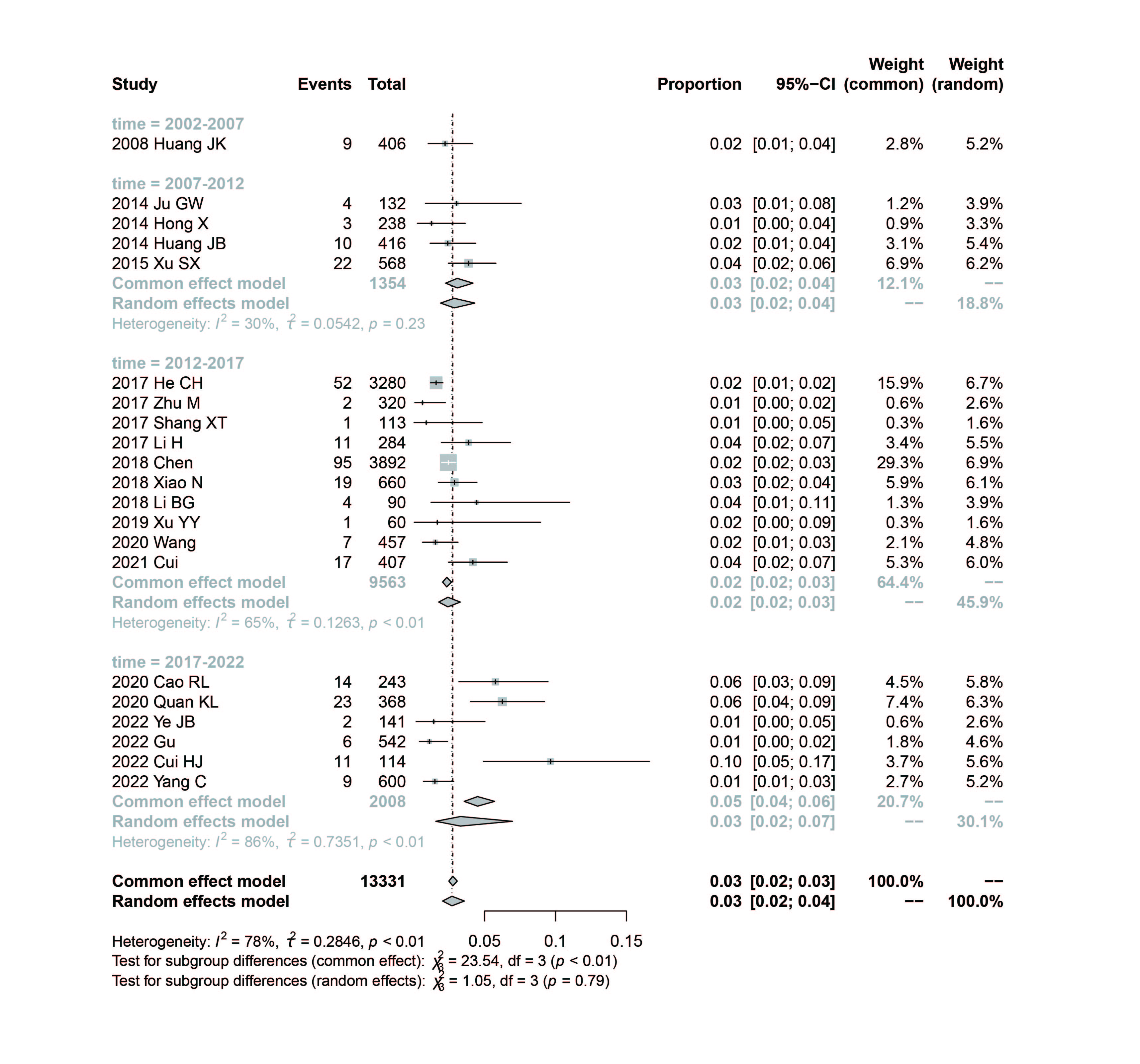

Supplement: Supplementary file 7 — Supplementary Material 7 [file 12894_2024_1415_MOESM7_ESM.jpg]

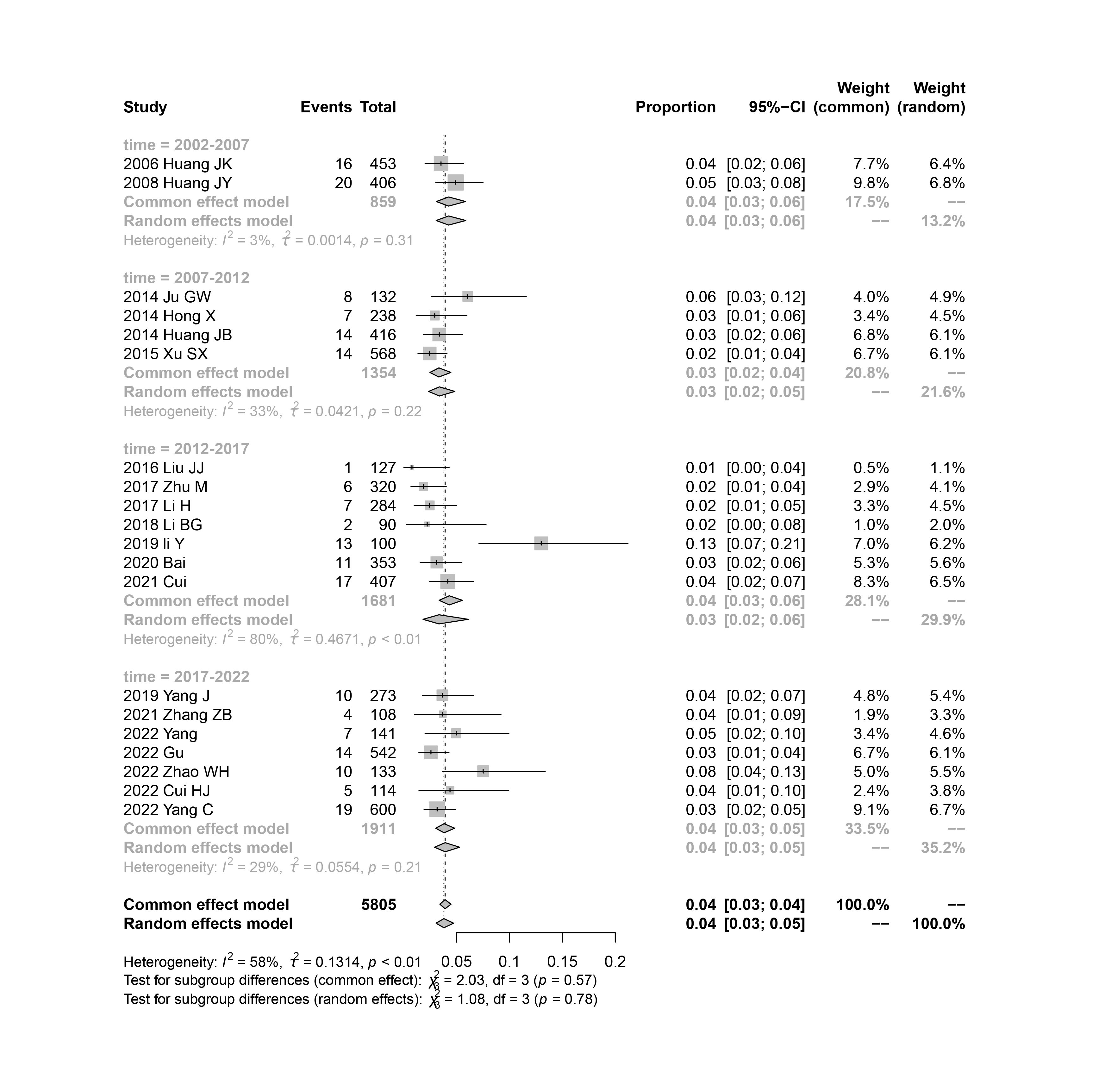

Supplement: Supplementary file 8 — Supplementary Material 8 [file 12894_2024_1415_MOESM8_ESM.jpg]

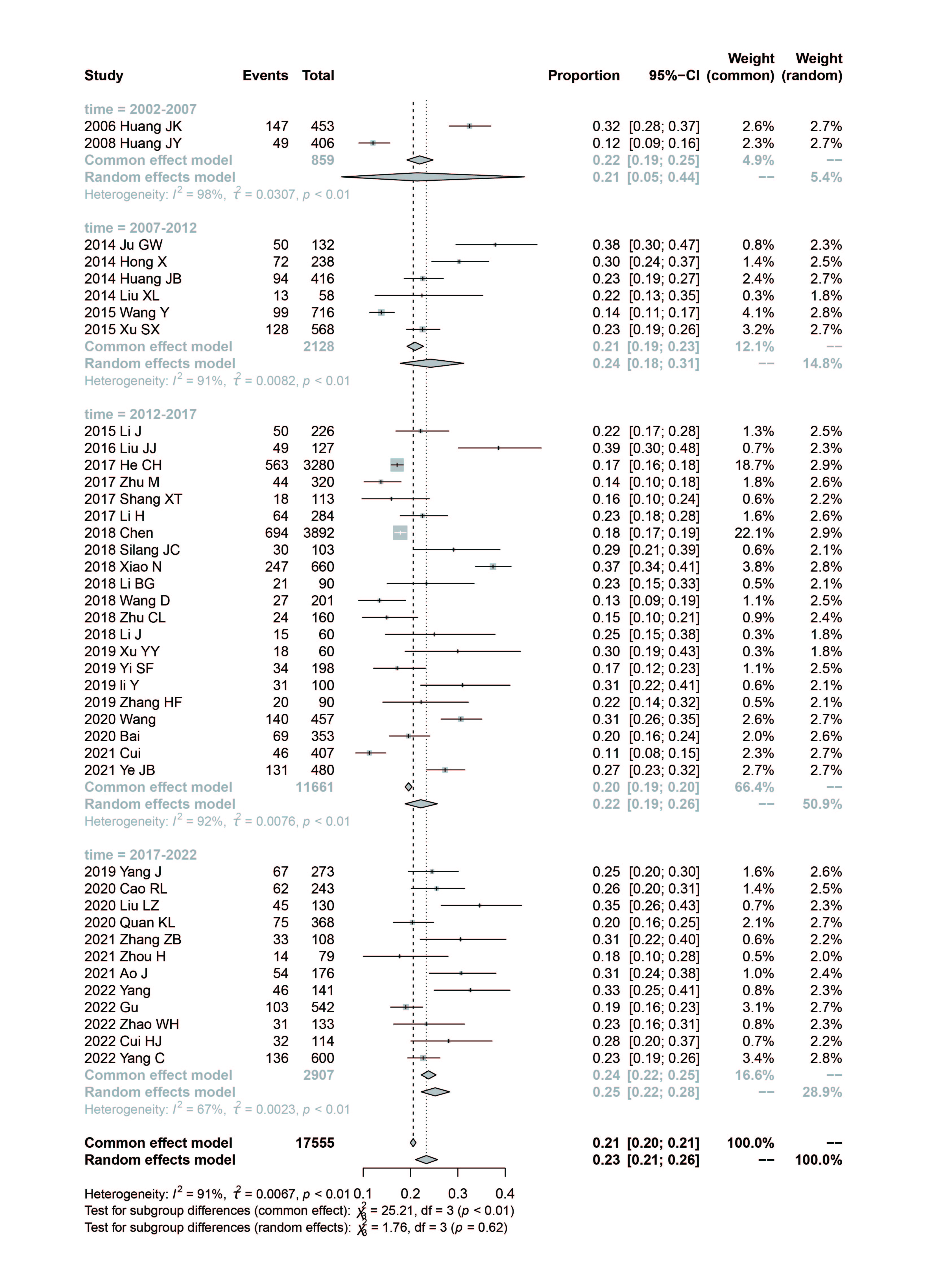

Supplement: Supplementary file 9 — Supplementary Material 9 [file 12894_2024_1415_MOESM9_ESM.jpg]

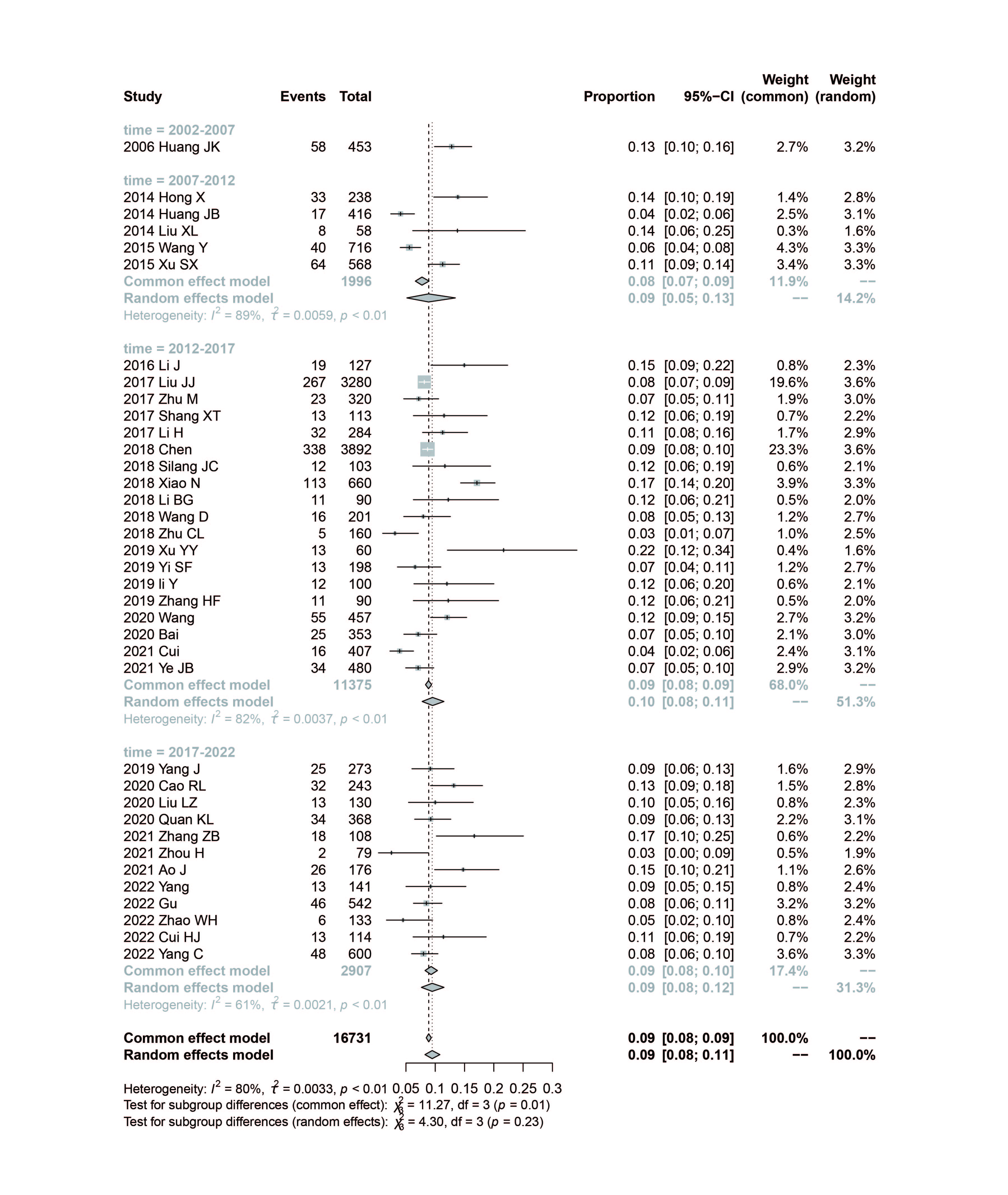

Supplement: Supplementary file 10 — Supplementary Material 10 [file 12894_2024_1415_MOESM10_ESM.jpg]

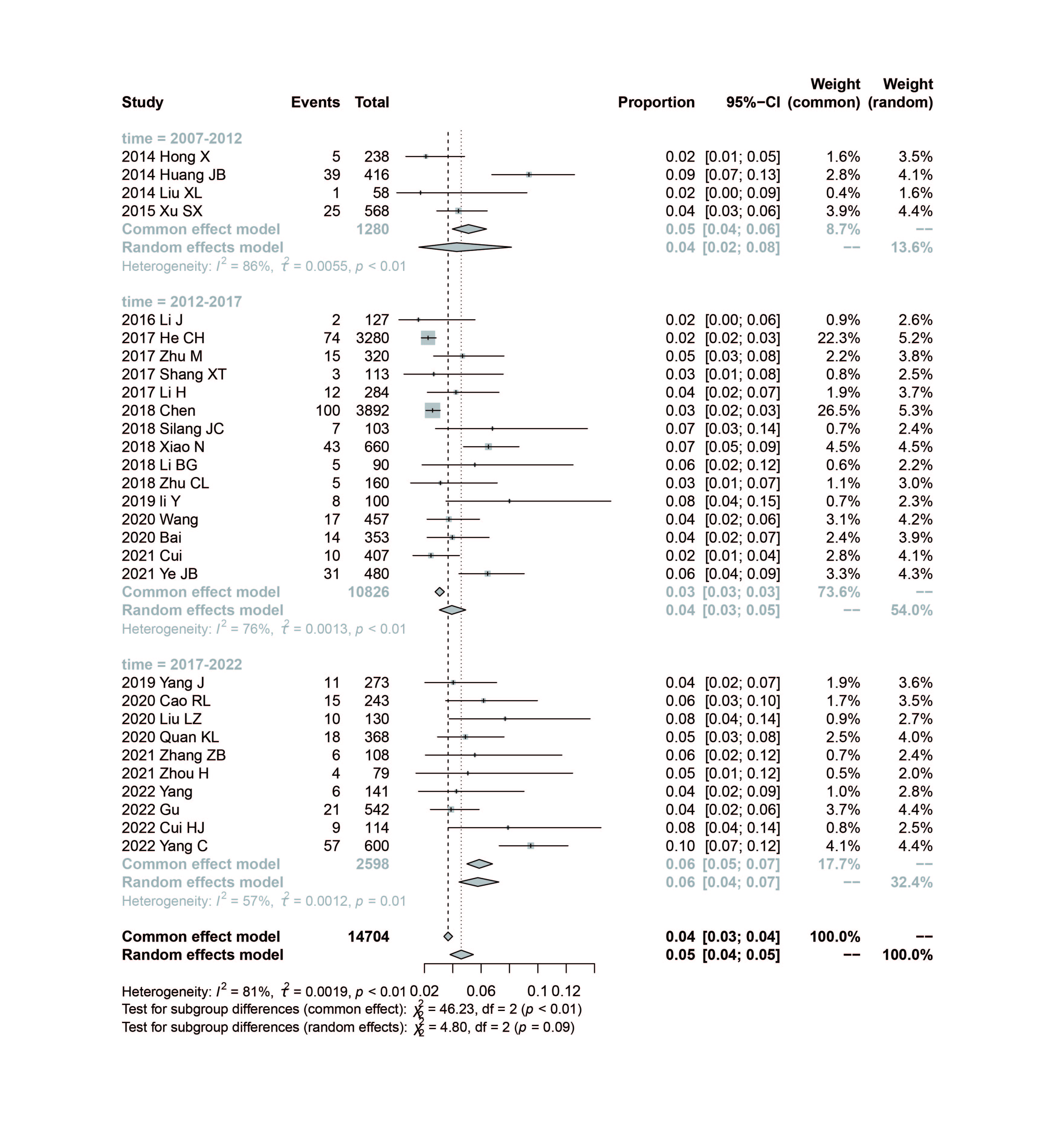

Supplement: Supplementary file 11 — Supplementary Material 11 [file 12894_2024_1415_MOESM11_ESM.jpg]

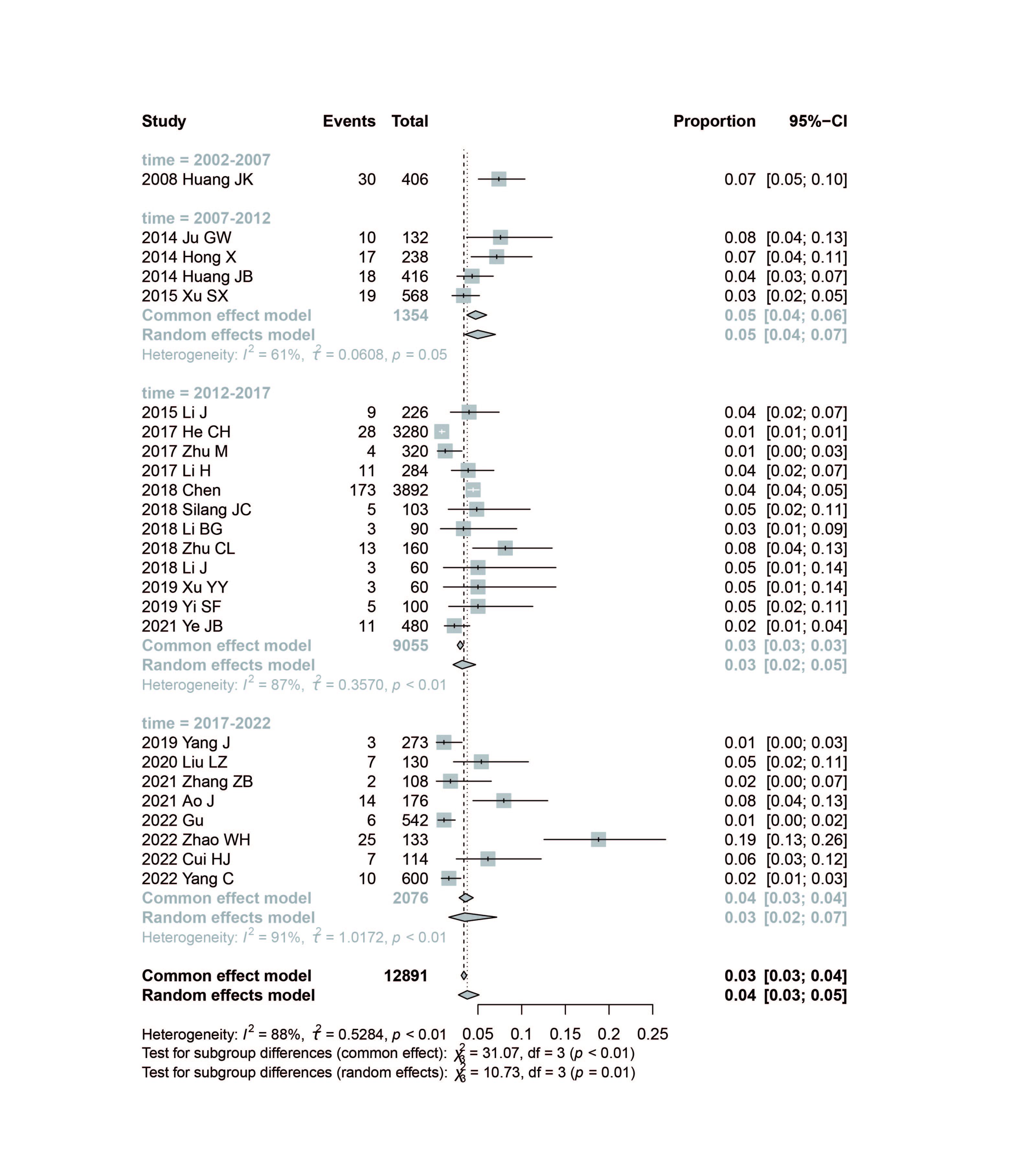

Supplement: Supplementary file 12 — Supplementary Material 12 [file 12894_2024_1415_MOESM12_ESM.jpg]

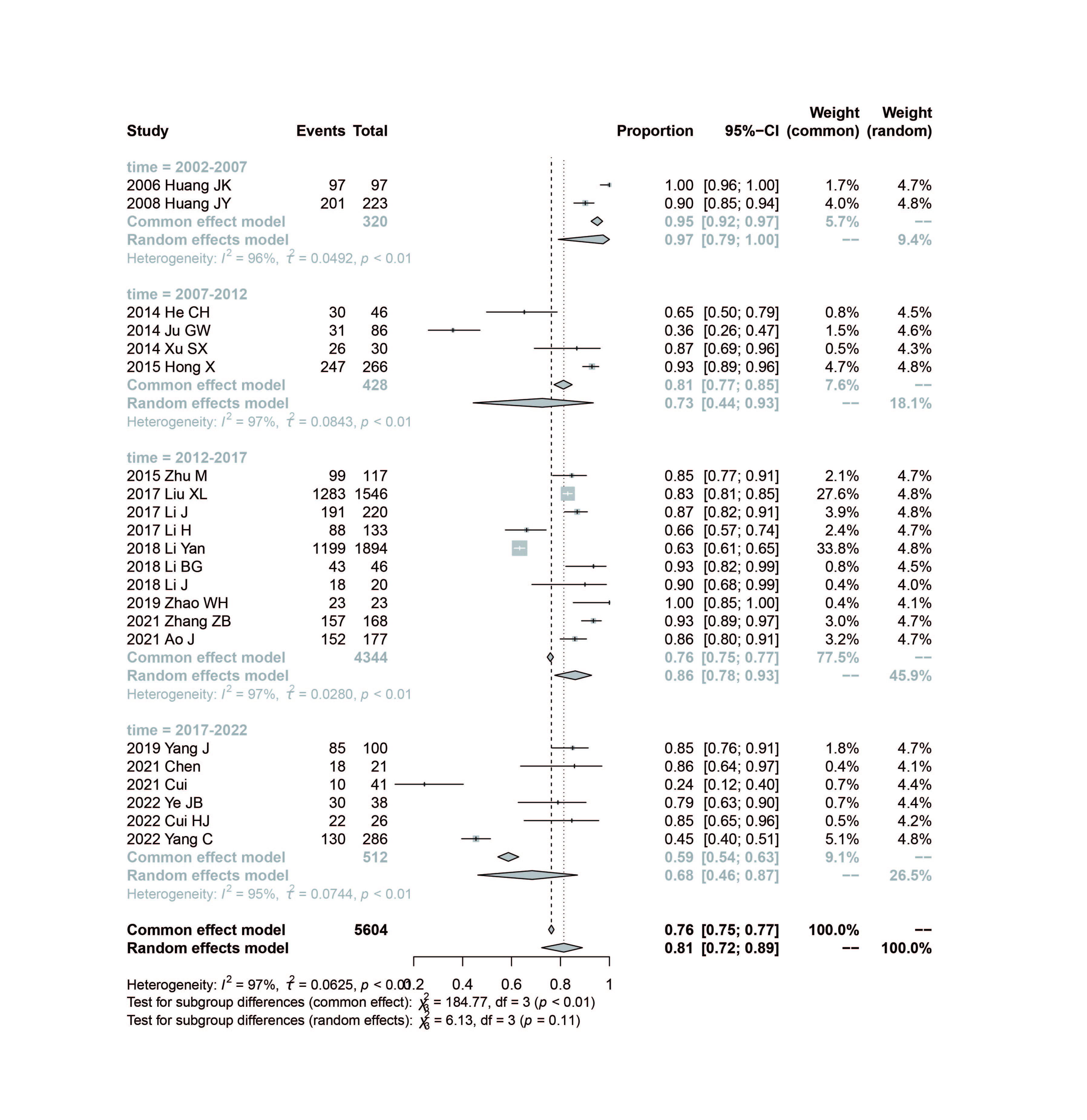

Supplement: Supplementary file 13 — Supplementary Material 13 [file 12894_2024_1415_MOESM13_ESM.jpg]

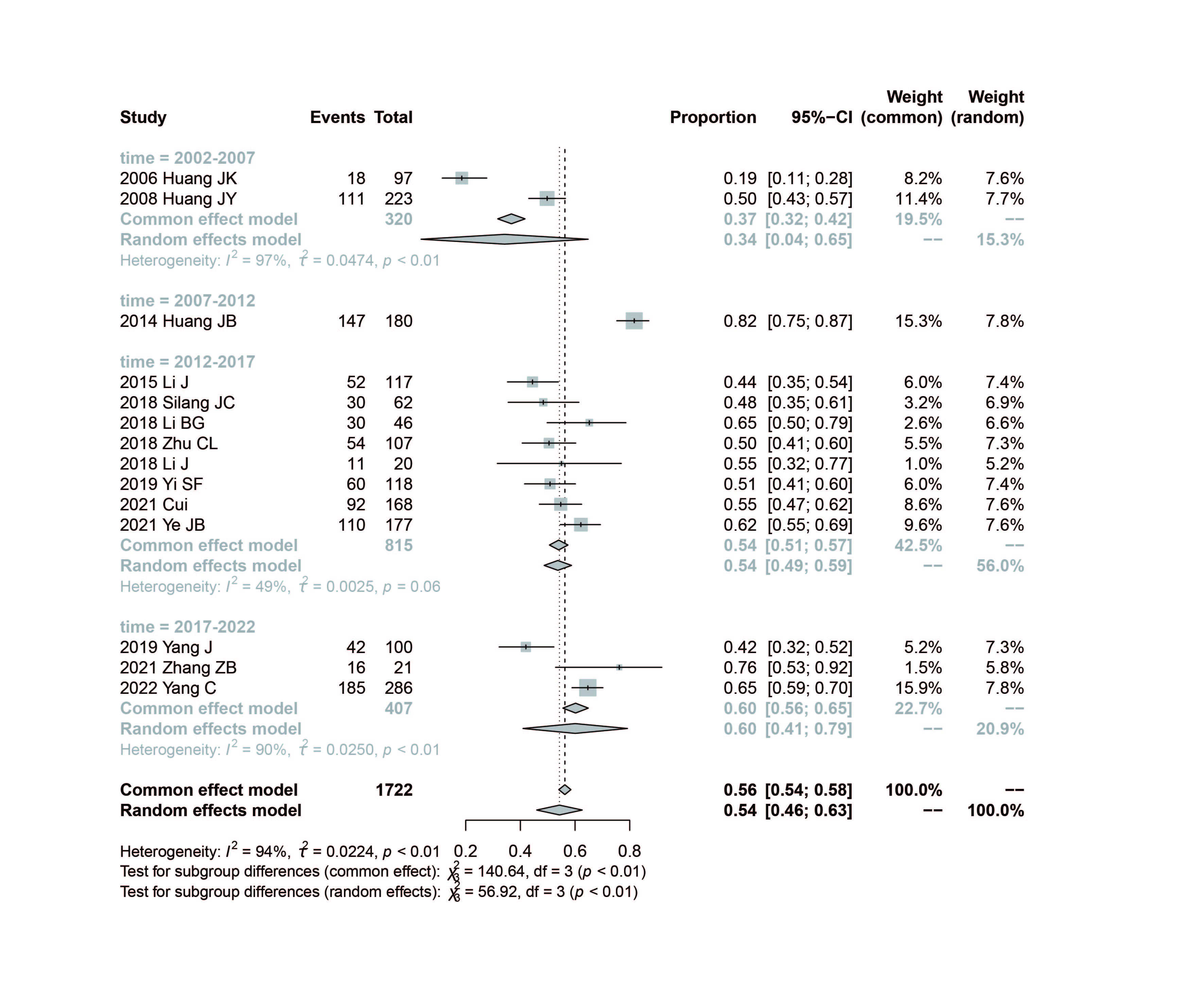

Supplement: Supplementary file 14 — Supplementary Material 14 [file 12894_2024_1415_MOESM14_ESM.jpg]

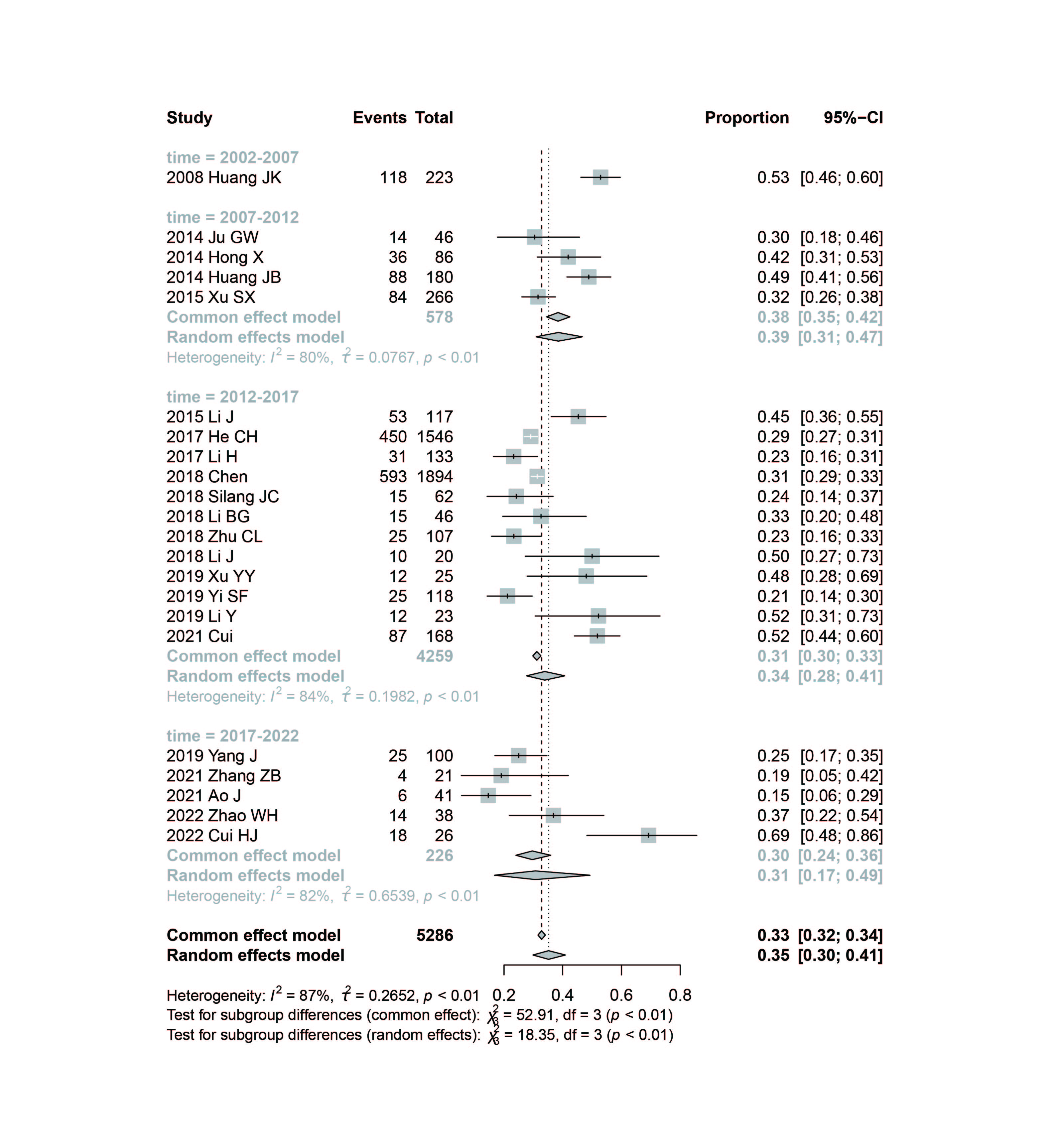

Supplement: Supplementary file 15 — Supplementary Material 15 [file 12894_2024_1415_MOESM15_ESM.jpg]

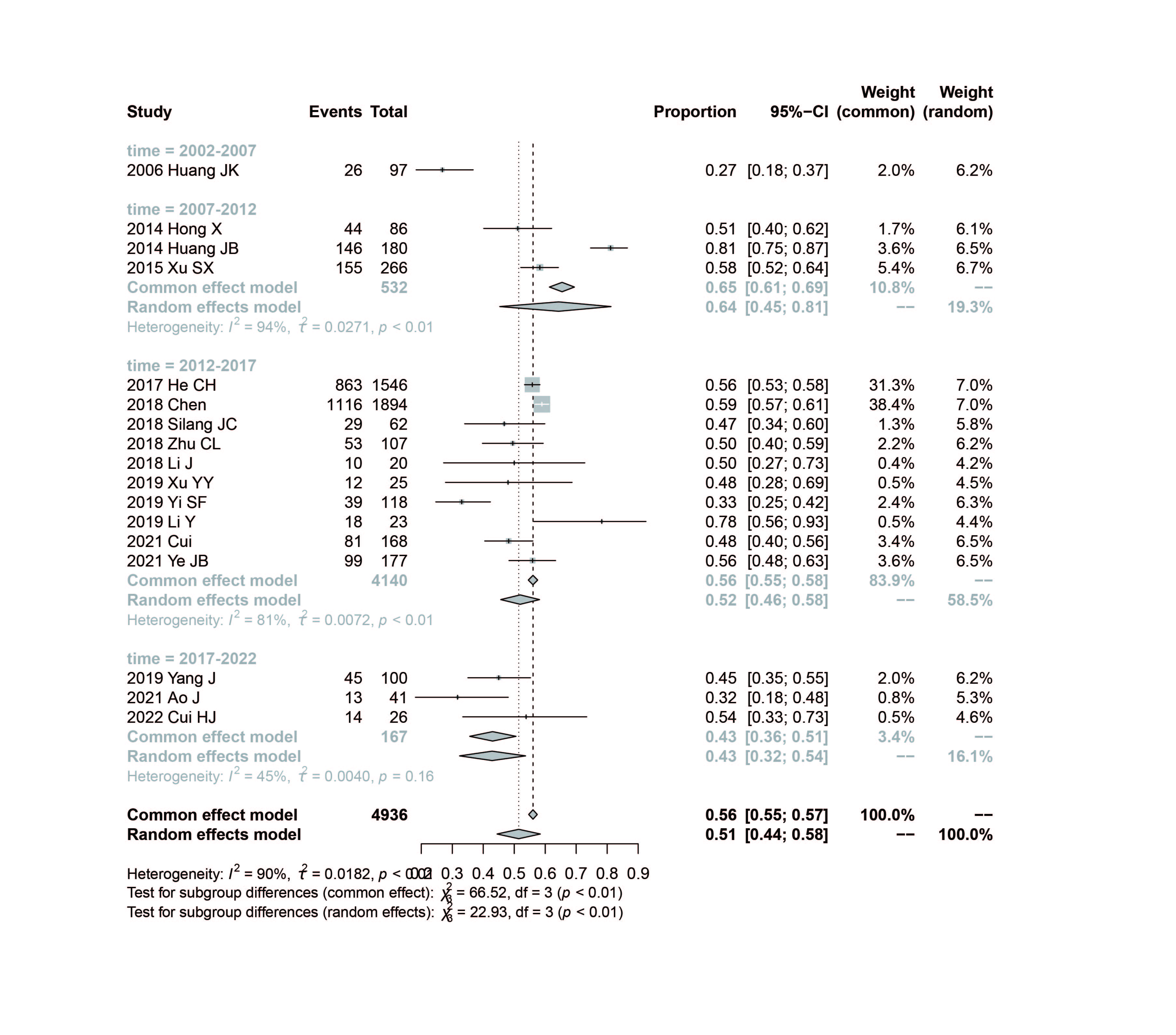

Supplement: Supplementary file 16 — Supplementary Material 16 [file 12894_2024_1415_MOESM16_ESM.jpg]

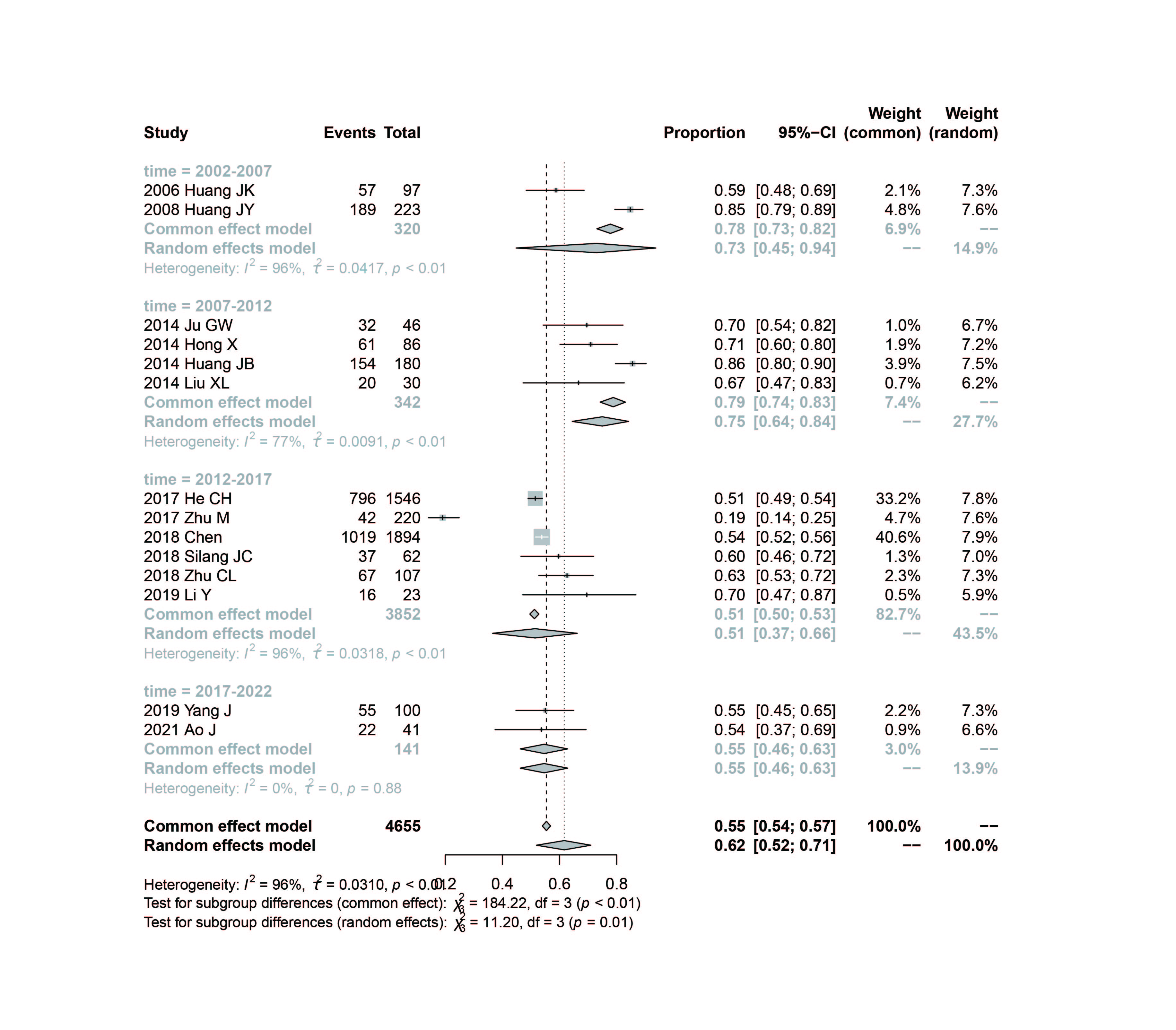

Supplement: Supplementary file 17 — Supplementary Material 17 [file 12894_2024_1415_MOESM17_ESM.jpg]

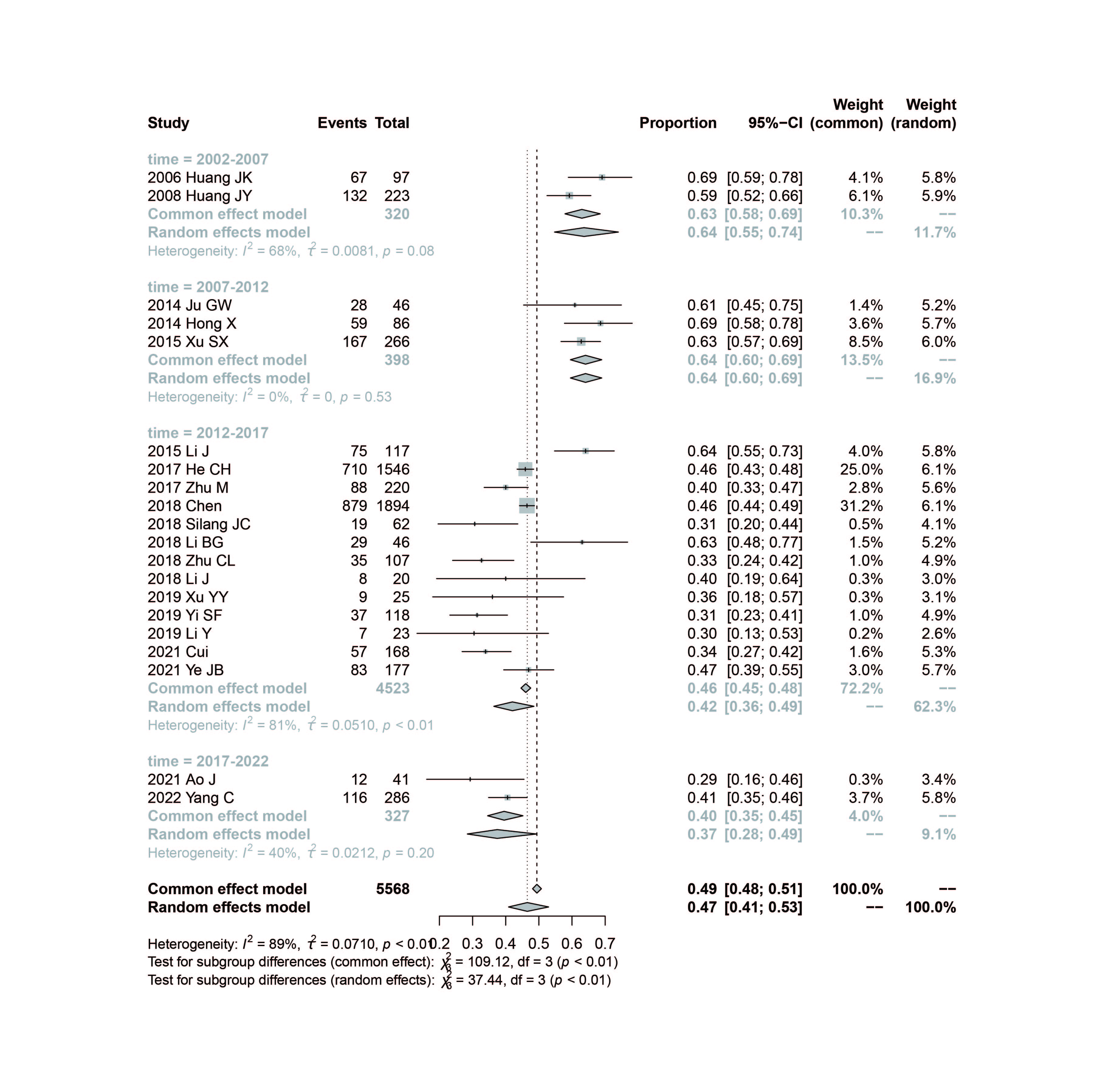

Supplement: Supplementary file 18 — Supplementary Material 18 [file 12894_2024_1415_MOESM18_ESM.jpg]

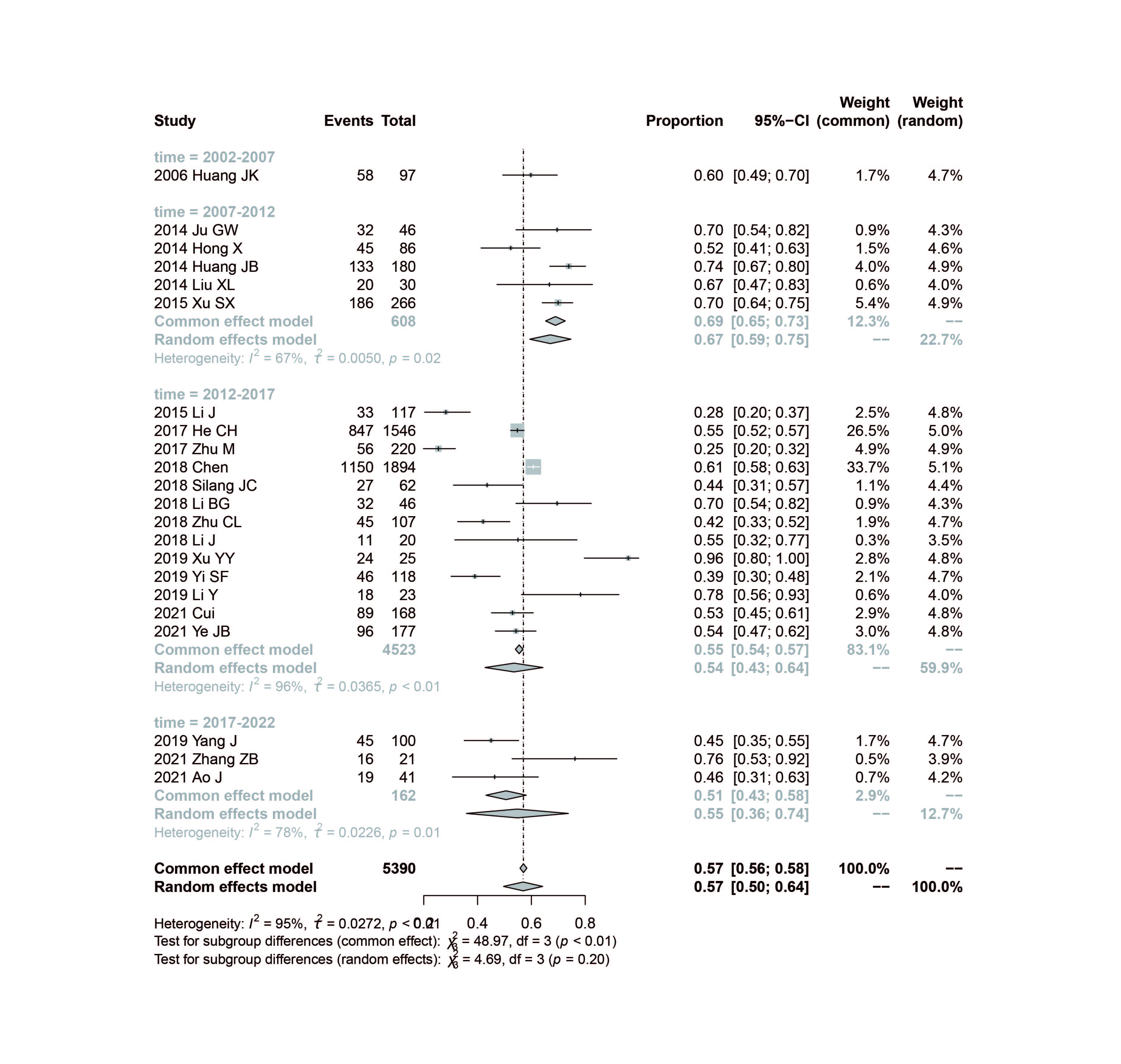

Supplement: Supplementary file 19 — Supplementary Material 19 [file 12894_2024_1415_MOESM19_ESM.jpg]

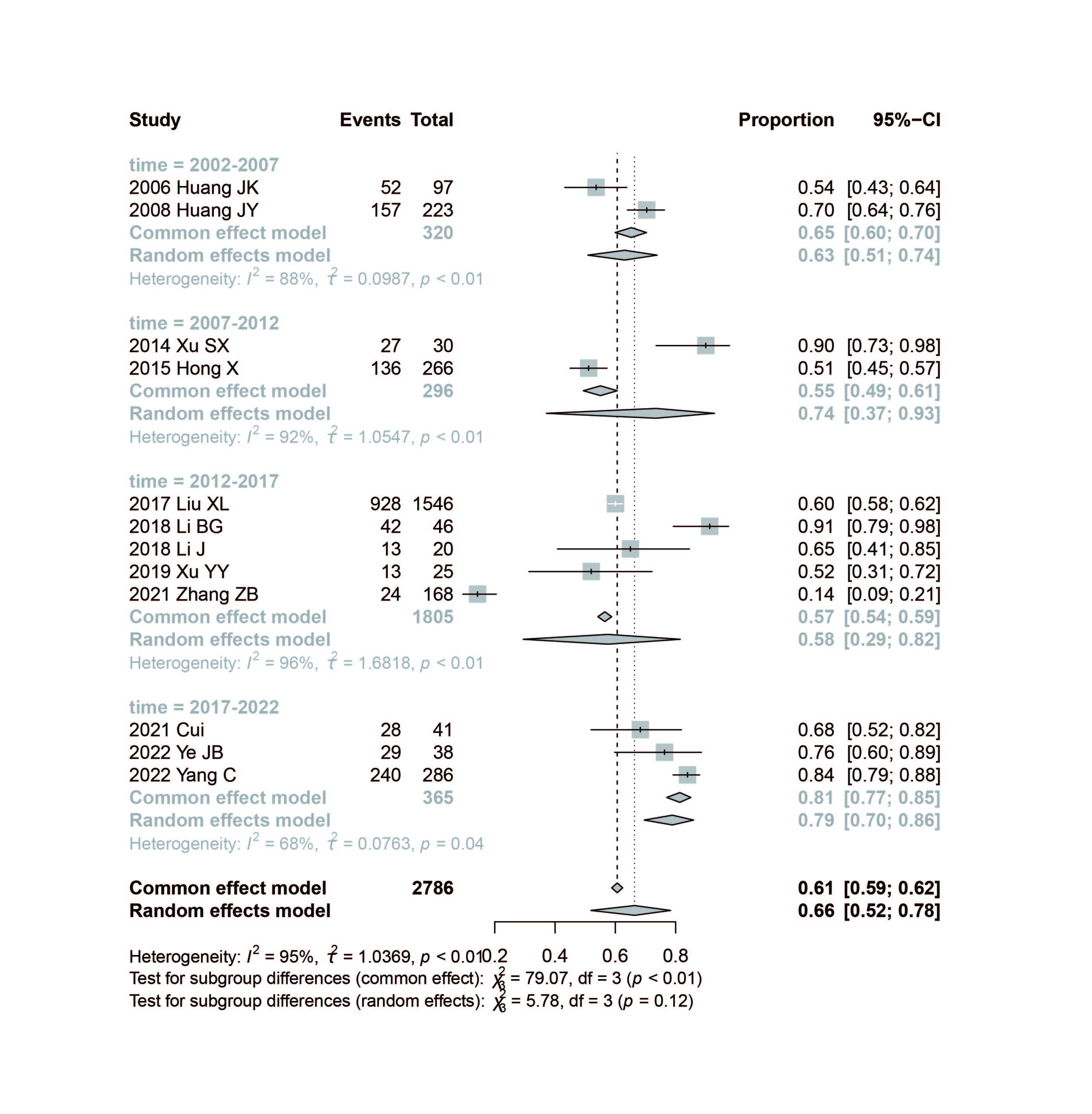

Supplement: Supplementary file 20 — Supplementary Material 20 [file 12894_2024_1415_MOESM20_ESM.jpg]

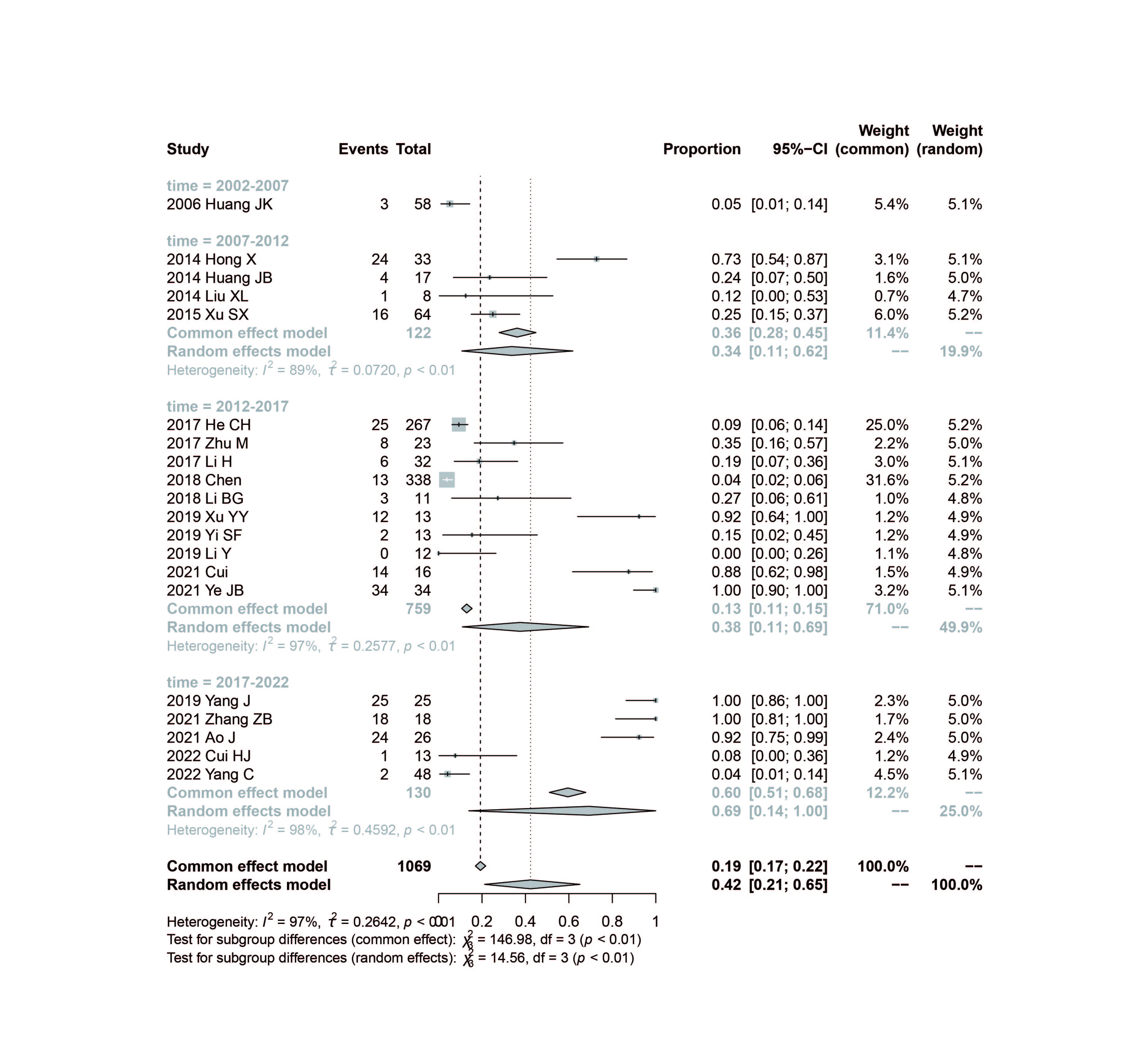

Supplement: Supplementary file 21 — Supplementary Material 21 [file 12894_2024_1415_MOESM21_ESM.jpg]

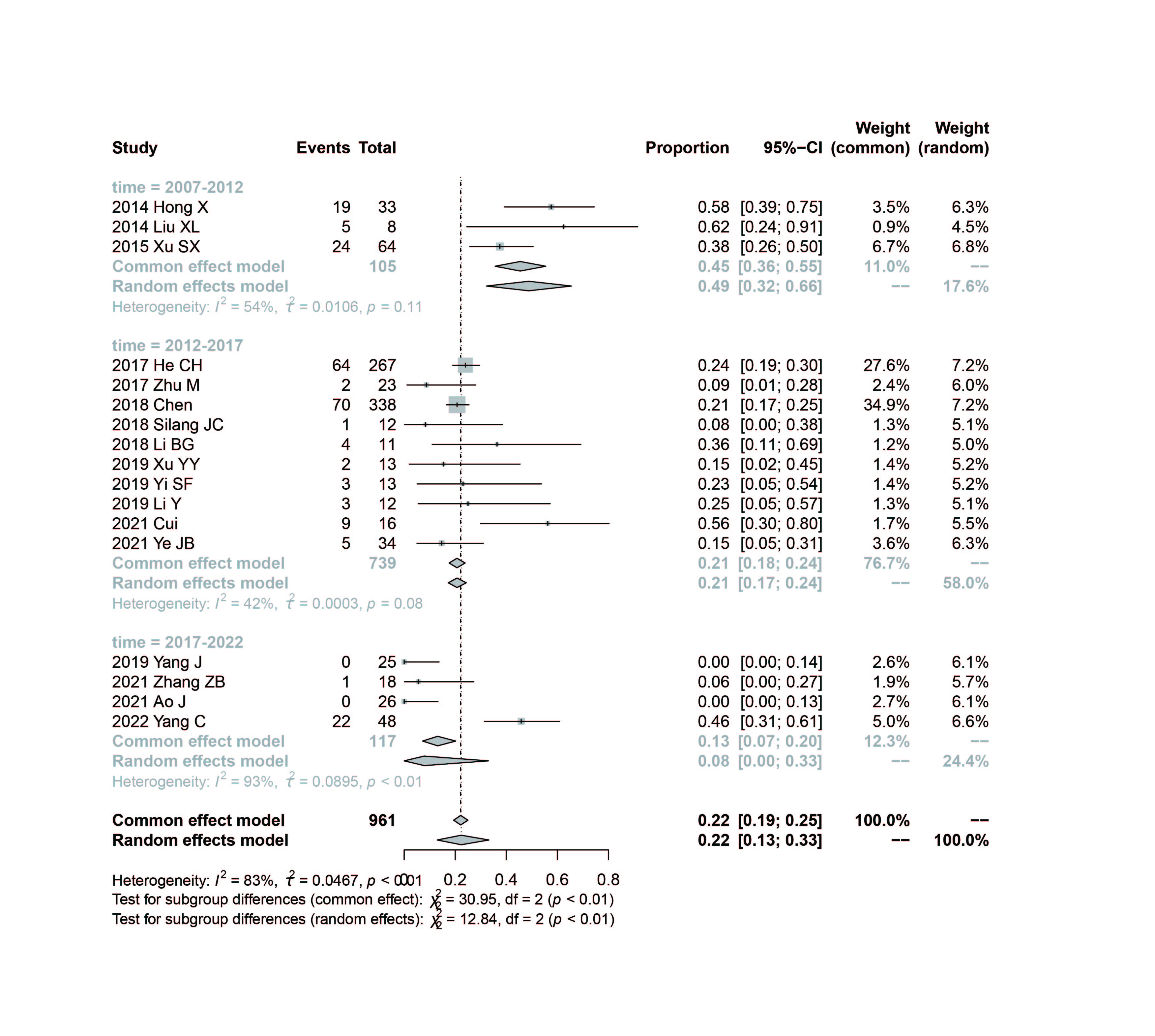

Supplement: Supplementary file 22 — Supplementary Material 22 [file 12894_2024_1415_MOESM22_ESM.jpg]

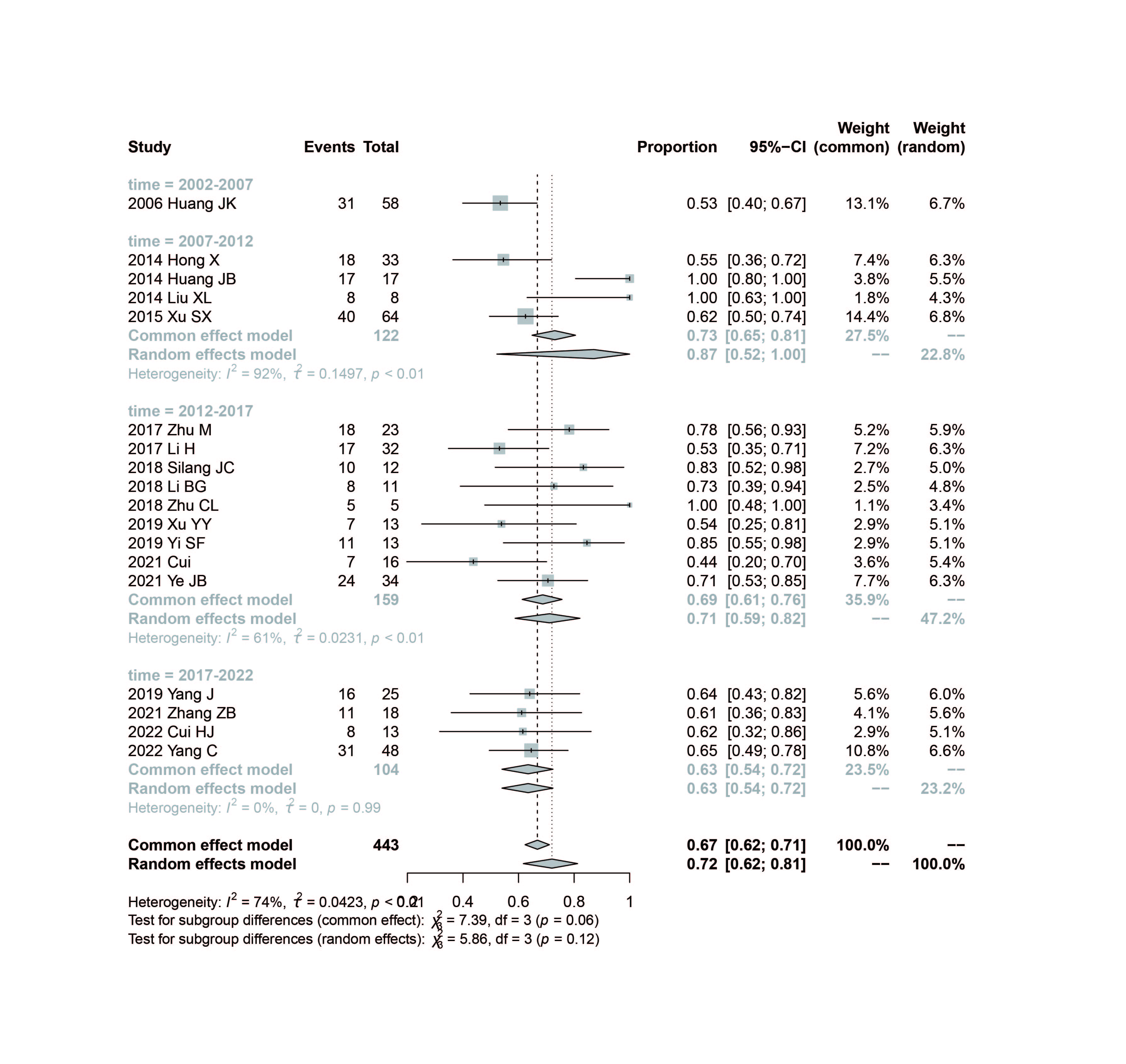

Supplement: Supplementary file 23 — Supplementary Material 23 [file 12894_2024_1415_MOESM23_ESM.jpg]

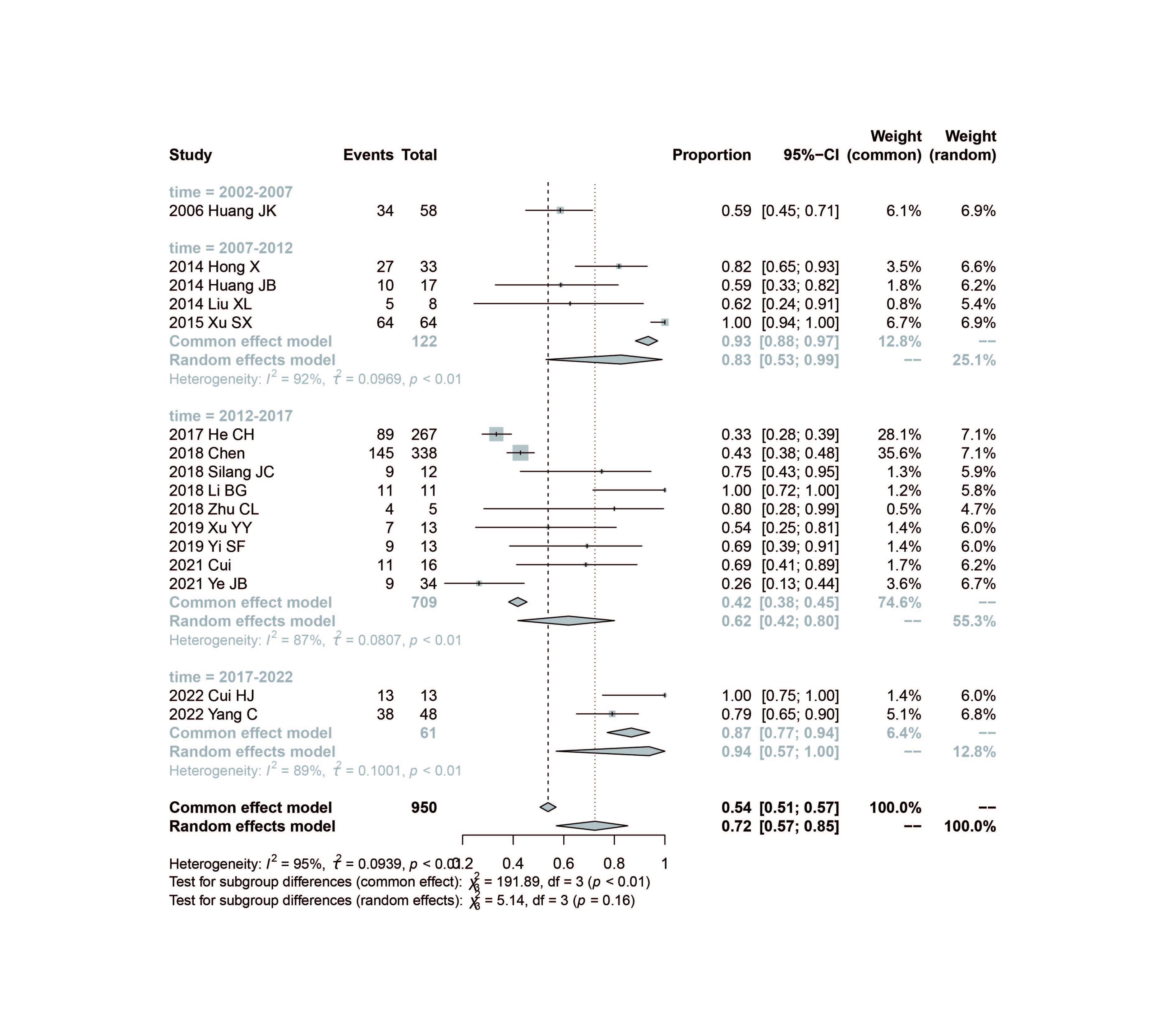

Supplement: Supplementary file 24 — Supplementary Material 24 [file 12894_2024_1415_MOESM24_ESM.jpg]

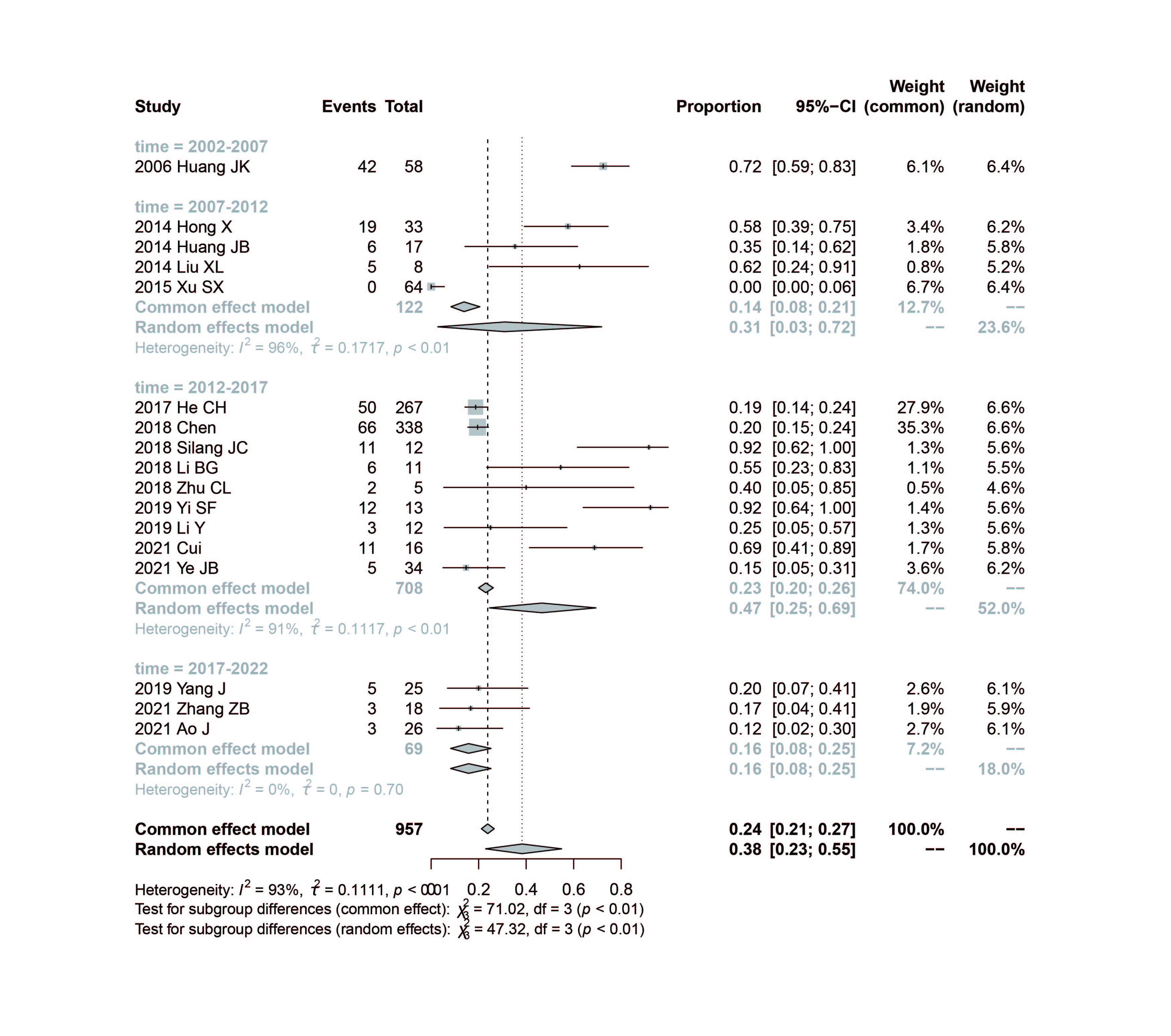

Supplement: Supplementary file 25 — Supplementary Material 25 [file 12894_2024_1415_MOESM25_ESM.jpg]

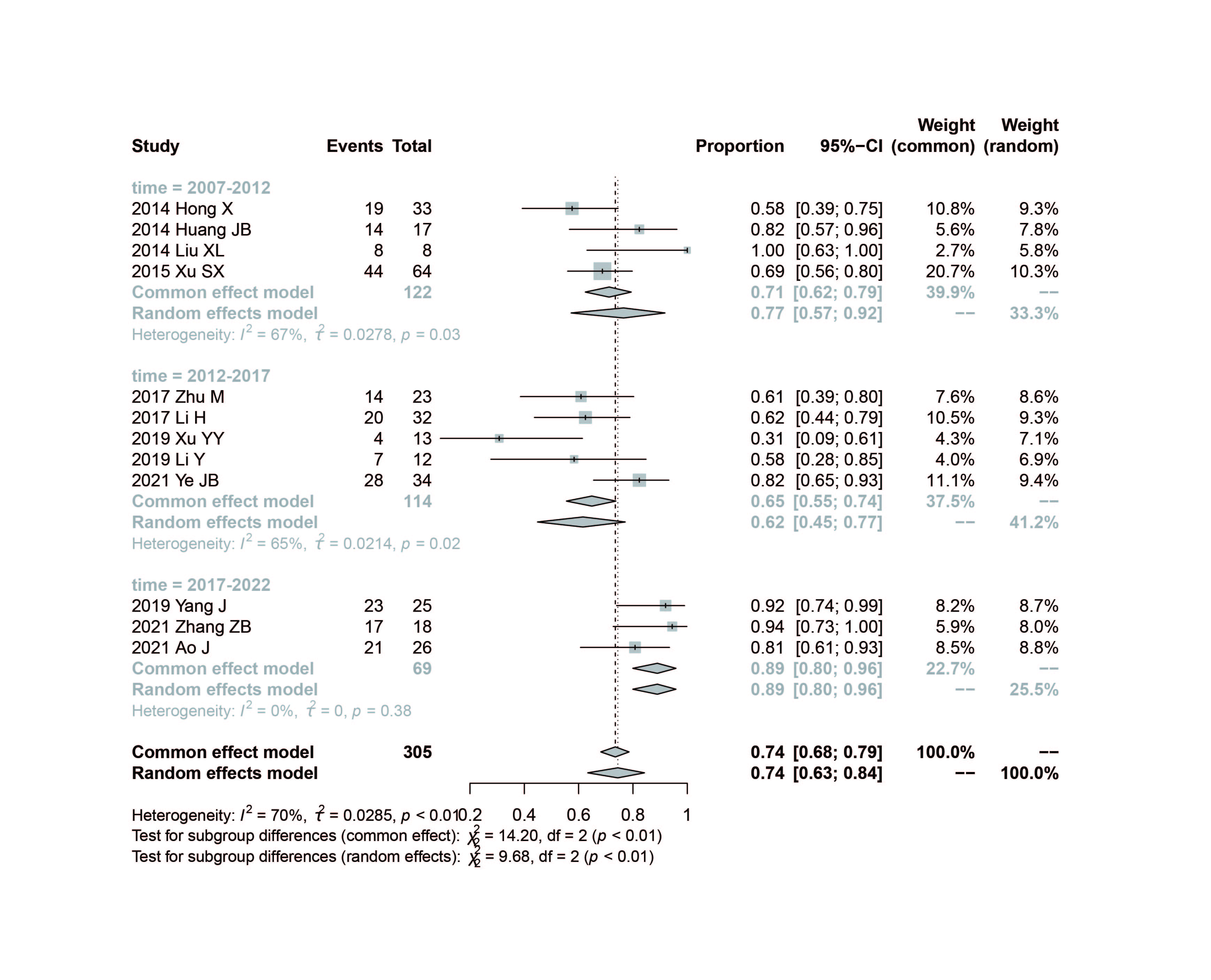

Supplement: Supplementary file 26 — Supplementary Material 26 [file 12894_2024_1415_MOESM26_ESM.jpg]
